# The impact of surgical delay on resectability of colorectal cancer: An international prospective cohort study

**DOI:** 10.1111/codi.16117

**Published:** 2022-04-24

**Authors:** COVIDSurg Collaborative, Michel Adamina, Adesoji Ademuyiwa, Adewale Adisa, Aneel A Bhangu, Ana Minaya Bravo, Miguel F Cunha, Sameh Emile, Dhruva Ghosh, James C Glasbey, Benjamin Harris, Debby Keller, Samuel Lawday, Hans Lederhuber, Sezai Leventoglu, Elizabeth Li, Maria Marta Modolo, Rohin Mittal, Helen M Mohan, Dmitri Nepogodiev, Marie Dione Parreño‐Sacdalan, Francesco Pata, Peter Pockney, Martin Rutegård, Neil Smart, Chris Varghese, James C Glasbey, Benjamin Harris, Aneel A Bhangu, Dmitri Nepogodiev, Kwabena Siaw‐Acheampong, Ruth A Benson, Edward Bywater, Daoud Chaudhry, Brett E Dawson, Jonathan P Evans, James C Glasbey, Rohan R Gujjuri, Emily Heritage, Conor S Jones, Sivesh K Kamarajah, Chetan Khatri, Rachel A Khaw, James M Keatley, Andrew Knight, Samuel Lawday, Elizabeth Li, Harvinder S Mann, Ella J Marson, Kenneth A McLean, Siobhan C Mckay, Emily C Mills, Gianluca Pellino, Maria Picciochi, Elliott H Taylor, Abhinav Tiwari, Isobel M Trout, Mary L Venn, Richard JW Wilkin, Aneel Bhangu, James C Glasbey, Neil J Smart, Ana Minaya‐Bravo, Jonathan P Evans, Gaetano Gallo, Susan Moug, Francesco Pata, Peter Pockney, Salomone Di Saverio, Abigail Vallance, Dale Vimalchandran, Ewen A Griffiths, Sivesh K Kamarajah, Richard PT Evans, Philip Townend, Keith Roberts, Siobhan McKay, John Isaac, Sohei Satoi, John Edwards, Aman S Coonar, Adrian Marchbank, Edward J Caruana, Georgia R Layton, Akshay Patel, Alessandro Brunelli, Samuel Ford, Anant Desai, Alessandro Gronchi, Marco Fiore, Max Almond, Fabio Tirotta, Sinziana Dumitra, Angelos Kolias, Stephen J Price, Daniel M Fountain, Michael D Jenkinson, Peter Hutchinson, Hani J Marcus, Rory J Piper, Laura Lippa, Franco Servadei, Ignatius Esene, Christian Freyschlag, Iuri Neville, Gail Rosseau, Karl Schaller, Andreas K Demetriades, Faith Robertson, Alex Alamri, Richard Shaw, Andrew G Schache, Stuart C Winter, Michael Ho, Paul Nankivell, Juan Rey Biel, Martin Batstone, Ian Ganly, Raghavan Vidya, Alex Wilkins, Jagdeep K Singh, Dinesh Thekinkattil, Sudha Sundar, Christina Fotopoulou, Elaine YL Leung, Tabassum Khan, Luis Chiva, Jalid Sehouli, Anna Fagotti, Paul Cohen, Murat Gutelkin, Rahel Ghebre, Thomas Konney, Rene Pareja, Rob Bristow, Sean Dowdy, TS Shylasree, Rajkumar Kottayasamy Seenivasagam, Joe Ng, Keiichi Fujiwara, Grant D Stewart, Benjamin Lamb, Krishna Narahari, Alan McNeill, Alexandra Colquhoun, John S McGrath, Steve Bromage, Ravi Barod, Veeru Kasivisvanathan, Tobias Klatte, Tom EF Abbott, Sadi Abukhalaf, Michel Adamina, Adesoji O Ademuyiwa, Arnav Agarwal, Murat Akkulak, Ehab Alameer, Derek Alderson, Felix Alakaloko, Markus Albertsmeier, Osaid Alser, Muhammad Alshaar, Sattar Alshryda, Alexis P Arnaud, Knut Magne Augestad, Faris Ayasra, José Azevedo, Brittany K Bankhead‐Kendall, Emma Barlow, David Beard, Ruth A Benson, Ruth Blanco‐Colino, Amanpreet Brar, Ana Minaya‐Bravo, Kerry A Breen, Chris Bretherton, Igor Lima Buarque, Joshua Burke, Edward J Caruana, Mohammad Chaar, Sohini Chakrabortee, Peter Christensen, Daniel Cox, Moises Cukier, Miguel F Cunha, Giana H Davidson, Anant Desai, Salomone Di Saverio, Thomas M Drake, John G Edwards, Muhammed Elhadi, Sameh Emile, Shebani Farik, Marco Fiore, J Edward Fitzgerald, Samuel Ford, Tatiana Garmanova, Gaetano Gallo, Dhruva Ghosh, Gustavo Mendonça Ataíde Gomes, Gustavo Grecinos, Ewen A Griffiths, Magdalena Gruendl, Constantine Halkias, Ewen M Harrison, Intisar Hisham, Peter J Hutchinson, Shelley Hwang, Arda Isik, Michael D Jenkinson, Pascal Jonker, Haytham MA Kaafarani, Debby Keller, Angelos Kolias, Schelto Kruijff, Ismail Lawani, Hans Lederhuber, Sezai Leventoglu, Andrey Litvin, Andrew Loehrer, Markus W Löffler, Maria Aguilera Lorena, Maria Marta Modolo, Piotr Major, Janet Martin, Hassan N Mashbari, Dennis Mazingi, Symeon Metallidis, Ana Minaya‐Bravo, Helen M Mohan, Rachel Moore, David Moszkowicz, Susan Moug, Joshua S Ng‐Kamstra, Mayaba Maimbo, Ionut Negoi, Milagros Niquen, Faustin Ntirenganya, Maricarmen Olivos, Kacimi Oussama, Oumaima Outani, Marie Dione Parreño‐Sacdalan, Francesco Pata, Carlos Jose Perez Rivera, Thomas D Pinkney, Willemijn van der Plas, Peter Pockney, Ahmad Qureshi, Dejan Radenkovic, Antonio Ramos‐De la Medina, Toby Richards, Keith Roberts, April C Roslani, Martin Rutegård, Juan José Segura‐Sampedro, Irène Santos, Sohei Satoi, Raza Sayyed, Andrew Schache, Andreas A Schnitzbauer, Justina O. Seyi‐Olajide, Neil Sharma, Catherine A Shaw, Richard Shaw, Sebastian Shu, Kjetil Soreide, Antonino Spinelli, Grant D Stewart, Malin Sund, Sudha Sundar, Stephen Tabiri, Philip Townend, Georgios Tsoulfas, Gabrielle H van Ramshorst, Raghavan Vidya, Dale Vimalachandran, Oliver J Warren, Duane Wedderburn, Naomi Wright, JI Valenzuela, C Alurralde, EL Caram, DG Eskinazi, R Badra, JS García, SM Lucchini, C Vasey, E Watson, J Cecire, S Salindera, A Sutherland, JH Ahn, S Chen, N Gauri, S Jang, F Jia, CS Mulligan, W Yang, G Ye, H Zhang, J Moss, T Richards, A Thian, UG Vo, K Bagraith, E Chan, D Ho, E Jeyarajan, S Jordan, GJ Nolan, M Von Papen, M Wullschleger, AC Dawson, A Drane, N Egoroff, J Gani, N Lott, P Pockney, D Phan, D Townend, C Bong, J Gundara, S Bowman, GR Guerra, N Gerns, A Riddell, NN Dudi‐Venkata, HM Kroon, T Sammour, D Mitchell, B Swinson, A Waldron, P Walker, AC Dawson, A Drane, EWY Lun, F Messner, D Öfner, K Emmanuel, M Grechenig, R Gruber, M Harald, T Jäger, L Öhlberger, J Presl, A Wimmer, İ Namazov, E Samadov, D Barker, R Boyce, A Doyle, A Eastmond, R Gill, M O’Shea, G Padmore, N Paquette, E Phillips, S St John, K Walkes, N Flamey, P Pattyn, W Ceelen, P Pattyn, D Van de Putte, Y Van Nieuwenhove, G Van Ramshorst, W Willaert, W Oosterlinck, J Van den Eynde, R Van den Eynde, S Aguiar Júnior, T Marques, P Camara, RK De Lima, E Della Giustina, PV Hoffmann, L Nacif, C Carvalho Ferro, GMA Gomes, I Lima Buarque, A Lira dos Santos Leite, L Pol‐Fachin, T Santos Bezerra, A Silva, D Silvestre, A Vieira Barros, G Laporte, M Salem, J Barakat Awada, TR Ijichi, NJ Kim, A Marreiro, B Muller, R Nunes, B Bodanese, JC Isoton, L Regina de Sampaio, C Vendrame, M Sokolov, P Gribnev, M Boutros, N Caminsky, G Ghitulescu, G Groot, A Persad, H Pham, M Wood, M Boutros, S Demyttenaere, R Garfinkle, C Brown, A Karimuddin, N Lee, J Liu, T Madani Kia, PT Phang, M Raval, K Tom, A Martel, C Nessim, J Stevenson, S Al Riyami, K Bali, D Bigam, K Dajani, A Dell, F Bellolio, N Besser, E Grasset, M Inzunza, M Quintana Martinic, C Riquoir Altamirano, M Ruiz Esquide, F Arias‐Amézquita, C Cétares, N Cortes Murgueitio, JL Gomez‐Mayorga, M Abadia, A Bonilla, H Facundo, O Guevara, DR Herrera Mora, LJ Jimenez Ramirez, E Manrique, RE Pinilla Morales, M Rey Ferro, BG Velasquez Cuasquen, G Bačić, D Karlović, D Kršul, M Zelić, B Bakmaz, I Ćoza, E Dijan, Z Katusic, J Mihanovic, I Rakvin, H Almezghwi, K Arslan, A Özant, N Özçay, K Frantzeskou, N Gouvas, G Kokkinos, P Papatheodorou, I Pozotou, O Stavrinidou, A Yiallourou, L Martinek, M Skrovina, I Szubota, M Peteja, J Žatecký, T Avlund, P Christensen, JL Harbjerg, LH Iversen, DW Kjaer, HØ Kristensen, M Mekhael, AL Ebbehøj, P Krarup, N Schlesinger, H Smith, A Crespo, P Díaz, R Rivas, N Tactuk, M El Kassas, W Omar, A Tawheed, M Talaat, A Abdelsamed, AY Azzam, H Salem, A Seleim, M AL Sayed, F Ashoush, E Elazzazy, E Essam, M Ewedah, E Hassan, M Metwalli, M Mourad, MS Qatora, A Sabry, A Samih, A Samir Abdelaal, S Shehata, K Shenit, D Attia, N Kamal, N Osman, S Alaa, HM Hamza, S M.elghazaly, MM Mohammed, MA Nageh, MM Saad, EA Yousof, A Eldaly, M Alrahawy, A Sakr, H Soliman, H Soltan, G Amira, I Sallam, M Sherief, A Sherif, G Ghaly, R Hamdy, A Morsi, H Salem, G Sherif, H Abdeldayem, I Abdelkader Salama, M Balabel, Y Fayed, Ahmed Elshawadfy Sherif, R Elmorsi, B Refky, K Bekele, JH Kauppila, E Sarjanoja, O Helminen, H Huhta, JH Kauppila, C Beyrne, L Jouffret, L Marie‐Macron, F Fredon, A Roux, Z Lakkis, S Manfredelli, A Chebaro, M El Amrani, K Lecolle, G Piessen, C Eveno, B Noiret, J Veziant, FR Pruvot, P Zerbib, Q Ballouhey, B Barrat, A Taibi, D Bergeat, A Merdrignac, B Le Roy, LO Perotto, A Scalabre, A Aimé, AC Ezanno, B Malgras, PA Bouché, S Tzedakis, E Cotte, O Glehen, L Bendjemar, H Braham, L Charre, N El Arbi, A Police, E Volpin, A D’Urso, D Mutter, B Seeliger, S Bonnet, C Denet, D Fuks, A Laforest, G Pourcher, A Seguin‐givelet, E Tribillon, E Duchalais, U Bork, J Fritzmann, C Praetorius, J Weitz, T Welsch, K Beyer, C Kamphues, J Lauscher, FN Loch, C Schineis, M Albertsmeier, A Kappenberger, T Schiergens, J Werner, R Becker, J Jonescheit, I Pergolini, D Reim, J Herzberg, H Honarpisheh, T Strate, C Boeker, I Hakami, JW Mall, K Nowak, T Reinhard, J Kleeff, C Michalski, U Ronellenfitsch, E Bertolani, A Königsrainer, MW Löffler, M Quante, C Steidle, L Überrück, C Yurttas, J Izbicki, C Nitschke, D Perez, FG Uzunoglu, P Antonakis, I Contis, D Dellaportas, A Gklavas, M Konstadoulakis, N Memos, I Papaconstantinou, A Polydorou, T Theodosopoulos, A Vezakis, MI Antonopoulou, DK Manatakis, N Tasis, N Arkadopoulos, N Danias, P Economopoulou, M Frountzas, P Kokoropoulos, A Larentzakis, N Michalopoulos, S Parasyris, J Selmani, T Sidiropoulos, P Vassiliu, K Bouchagier, S Klimopoulos, D Paspaliari, G Stylianidis, D Akrivou, K Baxevanidou, K Bouliaris, P Chatzikomnitsa, G Delinasios, C Doudakmanis, M Efthimiou, A Giaglaras, C Kalfountzos, C Kolla, G Koukoulis, K Zervas, S Zourntou, I Baloyiannis, A Diamantis, K Perivoliotis, G Tzovaras, P Christidis, O Ioannidis, L Loutzidou, IG Karaitianos, A Geroukalis, T Tsirlis, E Baili, A Charalabopoulos, T Liakakos, D Schizas, E Spartalis, A Syllaios, C Zografos, C Christou, V Papadopoulos, A Tooulias, G Tsoulfas, E Athanasakis, E Chrysos, I Tsiaoussis, S Xenaki, E Xynos, K Futaba, MF Ho, S Hon, TWC Mak, S Ng, CC Foo, B Banky, N Suszták, S Misra, P Pareek, JR Vishnoi, A Jain, S Mishra, T Mishra, JK Mitra, D Muduly, A Agrawal, PK Garg, R Kottayasamy Seenivasagam, KS Majumdar, N Mishra, MP Singh, S Chyau Patnaik, S Rao, P Reddy, RRR S, AR Saksena, J Y, R Ayloor Seshadri, PD Haque, P Jeyaraj, S Kannummal Veetil, A Mahajan, S Devarakonda, MR Jesudason, R Mittal, M Moorthy, H Yezzaji, M Aggarwal, P Dhamija, A Kumar, M Chisthi, G D, G George, VV Kollengode, KG Kuttanchettiyar, I Yadev, K Dharanipragada, R Kalayarasan, P Penumadu, L B, S Mathew, N Akhtar, A Chaturvedi, S Gupta, V Kumar, S Rajan, N Agrawal, A Arora, H Chaturvedi, M Jain, S Kumar, S Singh, GA Bhat, N Chowdri, A Mehraj, FQ Parray, ZA Shah, R Wani, Z Ahmed, MA Bhat, A Laharwal, M Mahmood, I Mir, Z Mohammad, J Muzamil, A Rashid, R Singh, A Ahmed, D Jain, A Pipara, A Desoouza, D Pandey, CS Pramesh, A Saklani, AA Islam, G Kembuan, H Pajan, M Aremu, A Canas‐Martinez, O Cullivan, C Murphy, P Owens, L Pickett, M Corrigan, A Daly, CA Fleming, P Jordan, MY Kayyal, S Killeen, N Lynch, S O’Brien, WAS Syed, L Vernon, A Hanly, H Heneghan, R Kennelly, ST Martin, D Winter, BA Fahey, J Larkin, P Mccormick, BJ Mehigan, H Mohan, P Shokuhi, J Smith, Y Bashir, G Bass, T Connelly, B Creavin, H Earley, JA Elliott, AE Gillis, D Kavanagh, P Neary, J O’Riordan, IS Reynolds, D Rice, PF Ridgway, M Umair, M Whelan, K Corless, L Finnegan, A Fowler, A Hogan, A Lowery, K McKevitt, E Nugent, ÉJ Ryan, JC Coffey, RM Cunningham, M Devine, D Nally, C Peirce, NP Hardy, PM Neary, S O’Malley, M Ryan, V Gaziants, R Gold‐Deutch, R Lavy, O Zmora, S Macina, NM Mariani, E Opocher, A Pisani Ceretti, F Bianco, M Marino, MV Marino, A Mirabella, G Vaccarella, G Sena, C Agostini, G Alemanno, I Bartolini, C Bergamini, A Bruscino, R De Vincenti, A Di Bella, L Fortuna, G Maltinti, P Muiesan, P Prosperi, MN Ringressi, M Risaliti, A Taddei, R Tucci, T Campagnaro, A Guglielmi, C Pedrazzani, S Rattizzato, A Ruzzenente, G Turri, DF Altomare, V Papagni, A Picciariello, P Bellora, G D’Aloisio, M Ferrari, E Francone, S Gentilli, H Nikaj, M Bianchini, M Chiarugi, F Coccolini, C Cremonini, G Di Franco, N Furbetta, D Gianardi, S Guadagni, L Morelli, S Musetti, M Palmeri, D Tartaglia, G Anania, P Carcoforo, M Chiozza, A De Troia, M Koleva Radica, M Portinari, MG Sibilla, A Urbani, N Fabbri, CV Feo, S Gennari, S Parini, E Righini, V Annessi, C Castro Ruiz, A Giunta, MT Montella, M Zizzo, U Grossi, S Novello, M Romano, S Rossi, G Zanus, G Esposito, F Frongia, A Pisanu, M Podda, C Belluco, A Lauretta, G Montori, L Moras, M Olivieri, CF Feo, T Perra, A Porcu, AM Scanu, A Aversano, F Carbone, P Delrio, K Di Lauro, A Fares Bucci, D Rega, G Spiezio, G Pirozzolo, A Recordare, C Vignotto, M Calabrò, F Farnesi, EG Lunghi, A Muratore, NS Pipitone Federico, G De Palma, G Luglio, G Pagano, FP Tropeano, L Baldari, L Boni, E Cassinotti, M Cosimelli, M Fiore, M Guaglio, L Sorrentino, A Agnes, S Alfieri, F Belia, A Biondi, V Cozza, D D’Ugo, V De Simone, F Litta, L Lorenzon, AA Marra, F Marzi, A Parello, R Persiani, C Ratto, F Rosa, O Scrima, G Sganga, A Belli, F Izzo, R Patrone, C Foppa, MM Carvello, F De Lucia, A Spinelli, A Aprile, P Batistotti, A Massobrio, D Pertile, S Scabini, D Soriero, A De Manzoni Garberini, F Mazzotti, F Pasini, G Ugolini, R Barone, SL Birolo, M Caccetta, A Deirino, M Garino, M Grasso, C Marafante, A Masciandaro, E Moggia, S Mungo, A Murgese, E Raggio, P Federico, P Maida, E Marra, G Marte, A Petrillo, P Tammaro, A Tufo, M Berselli, G Borroni, E Cocozza, L Conti, M Desio, A Rizzi, C Baldi, C Corbellini, GM Sampietro, P Bordoni, G Clarizia, F Fleres, M Franzini, A Grechi, A Longhini, A Spolini, E Baldini, P Capelli, L Conti, SM Isolani, M Ribolla, A Bondurri, F Colombo, L Ferrario, C Guerci, A Maffioli, T Armao, M Ballabio, P Bisagni, M Longhi, M Madonini, H Impellizzeri, M Inama, G Moretto, S Mochet, A Usai, F Bianco, P Incollingo, M Giacometti, S Zonta, L Marino Cosentino, A Sagnotta, LC Nespoli, N Tamini, A Anastasi, B Bartalucci, A Bellacci, G Canonico, L Capezzuoli, C Di Martino, P Ipponi, C Linari, M Montelatici, T Nelli, G Spagni, L Tirloni, A Vitali, E Abate, M Casati, L Laface, M Schiavo, A Arminio, A Cotoia, V Lizzi, F Vovola, R Vergari, S D’Ugo, N Depalma, MG Spampinato, A Annicchiarico, F Catena, M Giuffrida, G Perrone, G Baronio, F Carissimi, M Montuori, E Pinotti, G Brachini, A Chiappini, PM Cicerchia, B Cirillo, G De Toma, E Fiori, GB Fonsi, I Iannone, F La Torre, P Lapolla, S Meneghini, A Mingoli, P Sapienza, M Zambon, GT Capolupo, F Carannante, E Mazzotta, A Gattolin, M Migliore, R Rimonda, D Sasia, E Travaglio, A Chessa, A Fiorini, C Norcini, G Colletti, M Confalonieri, A Costanzi, C Frattaruolo, G Mari, M Monteleone, A Locatelli, C Riva, A Balconi, P De Nardi, P Parise, A Vignali, A Belvedere, P Bernante, M Droghetti, E Jovine, J Neri, D Parlanti, AP Pezzuto, G Poggioli, M Rottoli, IS Russo, M Tanzanu, T Violante, F Borghi, D Cianflocca, S Di Maria Grimaldi, D Donati, E Gelarda, G Giraudo, MC Giuffrida, A Marano, S Palagi, L Pellegrino, C Peluso, V Testa, F Agresta, D Prando, M Zese, G Armatura, A Frena, G Bertelli, P Marinello, F Notte, G Scotton, M Cervellera, A Gori, L Sartarelli, V Tonini, G Gallo, G Sammarco, G Vescio, F Di Marzo, D Rega, Delrio P, Y Kanemitsu, K Moritani, M Al Abdallah, F Ayasra, Y Ayasra, A Qasem, T Fahmawee, A Hmedat, K Obeidat, MK Abou Chaar, M Al‐Masri, H Al‐Najjar, F Alawneh, N Aldokali, O Senossi, MT Subhi, M Algallai, S Alwarfly, S Alzaede, M Gahwagi, M Moftah, D Burgan, E Kamoka, AI Kilani, M Abdelkabir, I Altomi, E Abdulwahed, E Alshareea, N Aribi, S Aribi, M Biala, R Ghamgh, A Alsoufi, I Ellojli, A Kredan, A Msherghi, K Alshareef, Ghadah Z Alkadiki, Faraj S Almaadany, S Bradulskis, E Dainius, E Kubiliute, J Kutkevičius, A Parseliunas, A Subocius, D Venskutonis, F Rasoaherinomenjanahary, JB Razafindrahita, LH Samison, CW Ngo, S Ramasamy, KH Hamdan, MdRazali.I, JA Tan, MR Thanapal, E Choong, RZM Lim, Nik Amin Sahid, F Hayati, J Jayasilan, RK Sriram, S Subramaniam, AF Ibrahim, A Che jusoh, AH Hussain, AS Mohamed Sidek, MF Mohd Yunus, JY Soh, MP Wong, AD Zakaria, Z Zakaria, ZA Mohd Azman, NQ Fathi, AC Roslani, R Xavier, MR Alvarez, F Cordera, R Hernandez, CE Soulé Martínez, Z Aboharp Hasan, LR Otoniel, EE Sosa Duran, J Melchor‐Ruan, E Romero Bañuelos, D Vilar‐Compte, GA Buerba, MÁ Mercado, OE Posadas‐Trujillo, N Salgado‐Nesme, C Sarre, L Amrani, A Benkabbou, B El Ahmadi, Y El Bouazizi, ZH Belkhadir, A Ghannam, AM Majbar, R Mohsine, A Souadka, R Hompes, EM Meima‐van Praag, AJM Pronk, S Sharabiany, BA Grotenhuis, L Hartveld, L Ebben, S Kuiper, J Melenhorst, M Poeze, L Posma‐Bouman, T Derksen, J Franken, S Oosterling, J Konsten, M Van Heinsbergen, A Adeyeye, E Enoch, SE Nwabuoku, TT Sholadoye, MA Tolani, J Olaogun, H Abiyere, O Babalola, A Okunlola, S Ali Sani, J Chinda, S Garba, P Mshelbwala, S Olori, A Olute, O Osagie, I Pius Ogolekwu, A Umar, LO Abdur‐Rahman, A Adeyeye, JO Bello, O Olasehinde, AA Popoola, J Abassy, K Ahmed, A Alvi, S Khan, A Pirzada, A Saleem, MT Siddiqui, K Turk, A Jamal, AA Kerawala, AS Memon, R Nafees Ahmed, L Rai, AA Ali, MF Afzal, MI Khokhar, B Ayub, P Ramesh, R Sayyed, M Ayyaz, U Butt, M Kashif, AU Qureshi, MW Farooka, A Ayubi, SH Waqar, I Al‐Slaibi, H I. A. Alzeerelhouseini, F Jobran, SA Abukhalaf, M Cukier, R Jocson, C Teh, E Uy Magadia, P Major, M Bąk, K Dubieńska, A Ławnicka, D Murawa, M Janik, P Kowalewski, A Kwiatkowski, R Roszkowski, P Sroczyński, M Walędziak, C Azevedo, D Machado, F Mendes, X De Sousa, U Fernandes, C Ferreira, G Guidi, A Marçal, R Marques, D Martins, R Vaz‐Pereira, B Vieira, JI Almeida, R Almeida‐Reis, T Correia de Sá, MJMA Costa, V Fernandes, I Ferraz, L Lima da Cruz, C Lima da Silva, L Lopes, N Machado, J Marialva, M Nunes Coelho, J Pedro, C Pereira, A Ribeiro, CG Ribeiro, R Santos, P Saraiva, R Silva, F Tavares, M Teixeira, AC Almeida, MJ Amaral, R Andrade, C Camacho, M Costa, A Lázaro, O Nogueira, A Oliveira, A Ruivo, M Silva, JFF Simões, V Devezas, F Jácome, J Nogueiro, A Pereira, H Santos‐Sousa, S Vaz, J Pinto, A Tojal, P Cardoso, R Martins, G Martins dos Santos, P Henriques, H Morais, S Sousa, N Cardoso, J Teixeira, R Pereira, T Revez, R Ribeiro, I Manso, J Domingues, E Amorim, VH Baptista, MF Cunha, II Sampaio da Nóvoa Gomes Miguel, JP Bandovas, N Borges, B Chumbinho, I Figueiredo de Barros, S Frade, J Gomes, A Kam da Silva Andrade, A Pereira Rodrigues, S Pina, N Silva, I Silveira Nunes, R Sousa, P Azevedo, B Costeira, C Cunha, R Garrido, P Miranda, M Peralta Ferreira, M Sousa Fernandes, J Azevedo, D Galvão, A Vieira, B Patrício, PMDD Santos, AC Vieira Paiva Lopes, R Cunha, A Faustino, A Freitas, JR Mendes, R Parreira, A Abreu da Silva, M Claro, D Costa Santos, AC Deus, JV Grilo, F Castro Borges, J Corte Real, S Henriques, MJ Lima, P Matos Costa, A Alagoa Joao, P Azevedo, R Camarneiro, I Capunge, M Fragoso, J Frazão, A Martins, V Pedro, R Pera, F Ramalho de Almeida, A Sampaio Soares, R Vale, M Vasconcelos, F Brito da Silva, A Caiado, F Fonseca, M Ângelo, JM Baião, D Martins Jordão, T Vieira Caroço, C Baía, R Canotilho, AM Correia, AP Ferreira Pinto, M Peyroteo, JF Videira, R Kassir, F Sauvat, E Bonci, V Gata, S Titu, C Bezede, A Chitul, E Ciofic, D Cristian, F Grama, C Ciubotaru, I Negoi, VM Negoita, B Stoica, O Ginghina, N Iordache, RV Iosifescu, M Mardare, RM Mirica, A Spanu, AB Văcăraș, M Zamfir‐Chiru‐Anton, P Tsarkov, I Tulina, A Abelevich, A Bazaev, AK Kokobelyan, A Yanishev, A Litvin, Y Litvina, A Provozina, M Agapov, T Garmanova, E Kazachenko, D Markaryan, E Galliamov, V Kakotkin, V Kubyshkin, E Semina, A Kamalov, A Novikova, A Zakharenko, M Alshahrani, F Alsharif, M Eskander, S Majrashi, A Mashat, M Alharthi, M Aljiffry, M Basendowah, N Malibary, M Nassif, A Saleem, A Samkari, N Trabulsi, MA Azab, D Alqahtani, H Jaloun, I Mudawi, S Al Awwad, M Alghamdi, T Alnumani, S Awad, M I Sharara, H Al Habes, M Alqannas, M Alyami, M Alzamanan, D Cortés‐Guiral, A Elawad, H Adi, F Al ahmad, A Al Ayed, Y Alishi, O AlAamer, N Alselaim, J Alfaifi, J D’Souza, K Al‐Khayal, N Alhassan, O Alobeed, S Alshammari, A Bin Nasser, T Bin Traiki, T Nouh, A Zubaidi, F Abdulfattah, E Al‐Kharashi, F Alanazi, F Albaqami, A Alghuliga, K Alsowaina, N Arab, F Badahdah, S Alobaysi, A Alshahrani, A Alzahrani, L Aleksić, A Antic, G Barisic, M Ceranic, Ž Grubač, J Jelenkovic, D Kecmanović, S Kmezić, D Knezevic, Z Krivokapic, S Latinčić, V Markovic, S Matić, M Miladinov, M Pavlov, I Pejovic, B Tadic, J Vasljević, D Velickovic, K Doklestic, P Gregoric, N Ivancevic, Z Loncar, D Micic, M Buta, A Cvetkovic, S Gacic, M Goran, N Jeftic, I Markovic, M Milanović, S Nikolic, L Pejnovic, N Savković, D Stevic, N Vucic, M Zegarac, A Karamarkovic, M Kenic, LJ Milic, B Kovacevic, I Krdzic, B Lieske, N Almgla, A Boutall, A Herman, C Kloppers, D Nel, S Rayamajhi, M Paniagua García Señorans, V Vigorita, E Acrich, E Baena Sanfeliu, O Barrios, T Golda, C Santanach, M Serrano‐Navidad, M Sorribas Grifell, RV Vives, Escolà D, A Jiménez, JA Alcázar, M Angoso‐Clavijo, F Blanco‐Antona, A Carabias‐Orgaz, R Díaz Maag, J Garcia, AGP García‐Plaza, JI Gonzalez‐Muñoz, L Muñoz‐Bellvis, JM Sánchez Tocino, AB Sanchez‐Casado, J Trebol, J Hernandez Gutierrez, A Tébar Zamora, P Palma, L Cayetano Paniagua, L Gomez Fernandez, P Collera Ormazabal, R Diaz del Gobbo, R Farre Font, R Flores Clotet, CJ Gómez Díaz, N Guàrdia, CA Guariglia, A Osorio Ramos, R Sanchez Jimenez, L Sanchon, C Soto Montesinos, L Alonso‐Lamberti, J García‐Quijada, J Jimenez Miramón, V Jimenez, JM Jover, R Leon, JL Rodriguez, A Salazar, A Valle Rubio, H Aguado López, R Bravo Infante, FB De Lacy, AM Lacy, A Otero, V Turrado‐Rodriguez, S Valverde, R Anula, R Avellana, E Camarero Rodríguez, V Catalán Garza, J Dziakova, M García Alonso, B Lasses Martínez, L López Antoñanzas, JM Muguerza, S Ochagavía, MJ Peña Soria, D Rivera‐Alonso, P Saez Carlin, C Sánchez del Pueblo, G Sanz Ortega, R Sanz‐López, A Torres, J Martín‐Arévalo, D Moro‐Valdezate, V Pla‐Marti, J Beltrán de Heredia, B De Andrés‐Asenjo, T Gómez Sanz, C Jezieniecki, H Nuñez Del Barrio, FJ Ortiz de Solórzano Aurusa, A Romero de Diego, M Ruiz Soriano, J Trujillo Díaz, A Vázquez Fernández, P Lora‐Cumplido, MV Sosa‐Rodríguez, A Galvan Perez, E Gonzalez‐Gonzalez, AM Minaya Bravo, C San Miguel, N Alonso de la Fuente, M Jimenez Toscano, EJ Grau‐Talens, B Martin‐Perez, JA Benavides Buleje, M Carrasco Prats, C Giménez Francés, JM Muñoz Camarena, PA Parra Baños, E Peña Ros, M Ramirez Faraco, M Ruiz‐Marín, M Valero Soriano, M Allue, P Colsa, M García Domínguez, T Gimenez Maurel, LF Martín Anoro, L Ponchietti, JM Rodriguez Artigas, M Roldón Golet, A Utrilla Fornals, M Estaire Gómez, Á Fernández Camuñas, EP Garcia Santos, E Jimenez Higuera, C Martínez‐Pinedo, V Muñoz‐Atienza, D Padilla‐Valverde, R Picón Rodríguez, S Sánchez‐García, D Sanchez‐Pelaez, C Curtis Martínez, L Sánchez‐Guillén, RC Colombari, E Del valle, M Fernández, P Lozano Lominchar, L Martín, C Rey Valcarcel, J Zorrilla Ortúzar, F Alcaide Matas, JM García Pérez, P Troncoso Pereira, JL Blas Laina, B Cros, J Escartin, J Garcia Egea, A Nogués, I Talal El‐Abur, C Yánez, I Mora‐Guzmán, M Achalandabaso Boira, R Sales Mallafré, H Marín, M Prieto Calvo, I Villalabeitia Ateca, U De Andres Olabarria, M Durán Ballesteros, FJ Fernández Pablos, FJ Ibáñez‐Aguirre, A Sanz Larrainzar, B Ugarte‐Sierra, MA Acosta Mérida, D Ortiz López, AF Yepes Cano, A Correa Bonito, L Delgado Búrdalo, M Di Martino, J García Septiem, R Maqueda González, E Martin‐Perez, P Calvo Espino, P Guillamot Ruano, L Colao García, D Díaz Pérez, E Esteban Agustí, P Galindo Jara, M Gutierrez Samaniego, MA Hernandez Bartolome, J Serrano González, A Alonso Poza, B Diéguez, M García‐Conde, M Hernández‐García, M Losada, E Alvarez, N Chavarrias, A Gegúndez Simón, S Gortázar de las Casas, J Guevara‐Martínez, MI Prieto Nieto, P Ramos‐Martín, I Rubio‐Perez, J Saavedra, A Urbieta, D Aparicio‐López, M Cantalejo diaz, MDC De Miguel Ardevines, MÁ Dobón Rascón, V Duque‐Mallén, I Gascon Ferrer, MT González‐Nicolás Trébol, C Gracia‐Roche, M Herrero Lopez, H Kälviäinen, A Martinez German, M Matute, N Sánchez Fuentes, MS Santero‐Ramirez, S Saudí, A Blazquez Martin, M Diez Alonso, P Hernandez, F Mendoza‐Moreno, E Ovejero Merino, C Vera Mansilla, B Matías‐García, A Quiroga‐Valcárcel, AG Barranquero, C Cerro Zaballos, J Núñez, J Ocaña, D Ramos, F Acebes García, M Bailon‐Cuadrado, AD Bueno Cañones, E Choolani Bhojwani, P Marcos‐Santos, T Miguel, D Pacheco Sánchez, B Pérez‐Saborido, J Sanchez Gonzalez, FJ Tejero‐Pintor, F Alconchel, A Conesa, J Gil Martínez, AI Gutiérrez Fernández, A Lopez Abad, T Nicolás‐López, P Ramirez Romero, MJ Roca Calvo, K Rodrigues, JJ Ruiz Manzanera, AI Soriano, A Cano, L Capitan‐Morales, J Cintas Catena, J Gomez‐Rosado, F Oliva Mompean, MA Pérez Sánchez, FD Río Lafuente, C Torres Arcos, J Valdes‐Hernandez, H Cholewa, M Frasson, C Martínez Chicote, J Sancho‐Muriel, B Estraviz, L Fernández Gómez Cruzado, M González de Miguel, A Landaluce‐olavarria, A Abad‐Gurumeta, A Abad‐Motos, E Martínez‐Hurtado, J Ripollés‐Melchor, A Ruiz‐Escobar, A Cuadrado‐García, L Garcia‐Sancho Tellez, J Heras Aznar, P Maté Mate, I Ortega Vázquez, AL Picardo, JA Rojo López, F Sanchez Cabezudo Noguera, D Serralta de Colsa, C Cagigas Fernandez, R Caiña Ruiz, M Gomez Ruiz, P Martínez‐Pérez, S Santarrufina Martinez, V Valbuena Jabares, EP Cagigal Ortega, I Cervera, P Díaz Peña, J Gonzalez, M Marqueta De Salas, M Perez Gonzalez, A Ramos Bonilla, L Rodríguez Gómez, M Alfonso Garcia, A Craus‐Miguel, L Fernández Vega, E Ferrer‐Inaebnit, A Gil Catalán, FX González Argente, S Jeri, A Oseira, N Pujol Cano, JJ Segura‐Sampedro, C Soldevila Verdeguer, B Villalonga, R Blanco‐Colino, E Espin‐Basany, G Pellino, S Srishankar, SPB Thalgaspitiya, A Arulanantham, GBKD Bandara, U Jayarajah, S Ravindrakumar, VSD Rodrigo, AA Ali karar, MHY Elhafiz, ME Adam Essa Adam, A Ahmed, M Saleh, S Arkani, J Freedman, E Angenete, J Park, A Älgå, G Heinius, M Nordberg, E Pieniowski, N Löfgren, M Rutegård, M Arigoni, M Bernasconi, D Christoforidis, M Di Giuseppe, D La Regina, F Mongelli, M Chevallay, O Dwidar, E Gialamas, M Sauvain, M Adamina, AS Crugnale, L Guglielmetti, G Peros, M Gass, J Metzger, A Scheiwiller, M Turina, T Al Asadi, S Alkhateb, R Altom, B Bakkar, S Maa Albared, S Melhem, A Hammed, S Hammed, MJ Kacem, H Maghrebi, A Sebai, A Aghayeva, I Hamzaoglu, I Sahin, E Akaydin, Z Aliyeva, E Aytac, B Baca, V Ozben, BB Ozmen, AE Arikan, IA Bilgin, H Kara, T Karahasanoglu, C Uras, A Akbas, Y Altinel, F Calikoglu, G Ercan, C Ercetin, NA Hacım, S Meriç, M Tokocin, T Vartanoglu, H Yigitbas, M Doğangün, N Iflazoğlu, Ö Yalkın, O Cennet, HA Dincer, T Erol, A Alhamed, S Ergün, MF Ozcelık, AN Sanli, SS Uludağ, M Velidedeoglu, AK Zengin, Y Kara, MA Bozkurt, A Kocatas, K Eyuboglu, A Guner, MA Usta, İF Azamat, E Balik, D Buğra, CB Kulle, SA Güler, A Güreşin, OC Tatar, NZ Utkan, A Yildirim, E Yüksel, A Abbasov, H Yanar, E Akin, F Altintoprak, G Cakmak, F Çelebi, H Demir, E Dikicier, N Firat, E Gönüllü, MB Kamburoğlu, IF Küçük, B Mantoglu, E Çolak, GO Kucuk, MS Uyanik, B Goksoy, E Bozkurt, E Capkinoglu, O Guven, M Mihmanli, S Omeroglu, M Tanal, G Yetkin, M Akalin, C Arican, EK Avci, C Aydin, S Demirli Atici, M Emiroglu, T Kaya, E Kebabçı, G Kilinc Tuncer, Y Kirmizi, H Öğücü, S Salimoğlu, İ Sert, C Tugmen, K Tuncer, G Uslu, D Yeşilyurt, A Yildiz, A Yildiz, FA Gultekin, H Lule, B Oguttu, J Agilinko, A Ahmeidat, M Bekheit, LK Cheung, BS Kamera, G Mignot, S Shaikh, P Sharma, A Al‐Mohammad, S Ali, J Ashcroft, O Baker, P Coughlin, RJ Davies, H Kyriacou, C Mitrofan, A Morris, W Raby‐Smith, SM Rooney, AA Singh, XS Tan, A Townson, E Tweedle, A Kattakayam, R Lunevicius, A Sheel, A Sud, M Sundhu, D Angelou, M Choynowski, B McAree, A McCanny, D Neely, F Kamel, L Kumar, R Madani, P Nisar, M Creanga, M Elniel, J Law, F Mosley, L Arrowsmith, W Campbell, T Grove, C Kontovounisios, O Warren, T Doulias, M Li, E Martin, H Rodwell, R Clifford, N Eardley, E Krishnan, N Manu, E Martin, S Roy Mahapatra, OL Serevina, C Smith, D Vimalachandran, K Emslie, PL Labib, G Minto, J Natale, P Panahi, LJ Rogers, A Abubakar, MM Akhter Rahman, E Chan, H O’Brien, K Sasapu, HJ Ng, A Day, A Hunt, N Laskar, A Gupta, J Steinke, S Thrumurthy, E Massie, K McGivern, D Rutherford, M Wilson, T Bacarese‐Hamilton, M Ip, A James, G Salerno, T Stockdale, S Handa, M Kaushal, A Kler, P Patel, J Redfern, S Tezas, Y Aawsaj, C Barry, L Blackwell, H Emerson, A Fisher, M Katory, A Mustafa, L Kretzmer, L Lalou, B Manku, I Parwaiz, J Stafford, M Abdelkarim, A Asqalan, T Gala, S Ibrahim, A Maw, R Mithany, R Morgan, G Sundaram Venkatesan, D Banfield, M Boal, O Brown, H Dean, AJ Boulton, CM Hardie, C McNaught, S Karandikar, D Naumann, F Chen, J Cheung, J Ayorinde, T Chase, T Cuming, A Ghanbari, L Humphreys, S Tayeh, A Aboelkassem Ibrahim, C Evans, H Ikram, M Loubani, S Nazir, A Robinson, T Sehgal, A Wilkins, J Dixon, M Jha, SV Thulasiraman, Y Viswanath, T Curl‐Roper, C Delimpalta, CCL Liao, V Velchuru, E Westwood, G Bond‐Smith, S Mastoridis, GD Tebala, C Verberne, N Anscomb, R Baldwin‐Smith, M Davies, C Grainger, A Haji, A Haq, JW Nunoo‐Mensah, M Rizk, MI Bhatti, H Boyd‐Carson, E Elsey, E Gemmill, P Herrod, M MohammedJibreel, E Lenzi, T Saafan, D Sapre, T Sian, N Watson, A Athanasiou, J Burke, F Costigan, H Elkadi, J Johnstone, C Nahm, S Annamalai, C Ashmore, A Kourdouli, CS Chean, S Dharamavaram, N Kulkarni, I Pereira, K Shanthakunalan, B Srikumar, A Askari, N Cirocchi, S Kudchadkar, K Patel, J Sagar, R Talwar, M Abdalla, O Ismail, K Newton, N Stylianides, A Aderombi, O Bajomo, K Beatson, WV Garrett, V Ng, R Al‐Habsi, GS Divya, F Dixon, BD Keeler, RJ Egan, I Fabre, R Harries, Z Li, K Parkins, N Spencer, D Thompson, C Gemmell, C Grieco, L Hunt, F Mahmoud Ali, K Seebah, I Shaikh, L Sreedharan, M Youssef, J Shah, M Baguley, B Heer, M Rogers, R Woods, SJ Mills, K Sahnan, ME Ahmed, SI Bukhari, B Illingworth, S Kanthasamy, E Knights, SL Ong, R Pujari, KHM Tan, R Vanker, M Michel, S Patil, S Ravindran, J Sarveswaran, L Scott, J Khan, A Bhangu, LD Cato, M Kamal, R Kulkarni, A Parente, S Saeed, D Vijayan, C English, J Evans, A Fell, C Halkias, R Merh, S Nikolaou, S Kaul, AH Khan, F Khan, S Mukherjee, M Patel, M Sarigul, S Singh, A Adiamah, H Brewer, A Chowdhury, J Evans, D Humes, J Jackman, A Koh, C Lewis‐Lloyd, O Oyende, J Reilly, D Worku, C Bisset, S Moug, R Chadha, S Math, I Sarantitis, S Timbrell, L Vitone, G Faulkner, G Brixton, L Findlay, A Majkowska, J Manson, R Potter, A Bhalla, Z Chia, P Daliya, E Grimley, FL Malcolm, E Theophilidou, IR Daniels, GE Fowler, LH Massey, FD McDermott, N Rajaretnam, N Angamuthu, S Chowdhury, J Gilliland, C Hart, J Knowles, R Mirnezami, M Varcada, AJ Beamish, D Magowan, H Nassa, C Price, L Smith, F Solari, AM Tang, G Williams, E Davies, P Hawkin, T Raymond, O Ryska, R Baron, S Gahunia, F McNicol, J Russ, P Szatmary, A Thomas, JD Jayasinghe, C Knowles, FS Ledesma, A Minicozzi, L Navaratne, R Ramamoorthy, C Sohrabi, MA Thaha, M Venn, R Atherton, M Brocklehurst, J McAleer, E Parkin, A Aladeojebi, M Ali, A Gaunt, C Hammer, J Stebbing, E Coomber, O Williams, J Bunni, K Fairhurst, S Mitchell, S Richards, I Hraishawi, C McIlmunn, S McIntosh, S Bhasin, AS Bodla, A Burahee, A Crichton, R Fossett, N Yassin, C Barlow, D Ding, J Foster, L Longstaff, SR Brown, M Lee, T Newman, C Steele, A Baker, C Konstantinou, S Ramcharan, RJW Wilkin, HV Colvin, Z Shakoor, S Lawday, A Lyons, S Newman, E Chung, R Hagger, A Hainsworth, A Karim, H Owen, A Ramwell, K Williams, J Hall, G Harris, T Royle, LJ Watson, P Asaad, B Brown, S Duff, A Khan, F Moura, B Wadham, S Mccluney, C Parmar, S Shah, MS Babar, S Goodrum, H Whitmore, D Balasubramaniam, B Jayasankar, S Kapoor, A Ramachandran, N Beech, M Chand, L Green, H Kiconco, R McEwen, J Pereca, S Arumugam, B Ibrahim, K Khan, K Gash, L Gourbault, TA Maccabe, C Newton, M Baig, H Bates, N Dunne, A Khajuria, V Ng, DR Sarma, T Shortland, N Tewari, MA Akhtar, A Brunt, J McIntyre, K Milne, MM Rashid, A Sgrò, KE Stewart, A Turnbull, G Gossedge, S O’Donnell, F Oldfield, M Aguilar Gonzalez, S Talukder, P Eskander, M Hanna, J Olivier, P Basnyat, H Davis, P Montauban, A Shrestha, C Magee, S Powell, I Flindall, A Hanson, V Mahendran, S Green, M Lim, L MacDonald, V Miu, L Onos, K Sheridan, R Young, F Alam, O Griffiths, C Houlden, VS Kolli, AK Lala, Z Seymour, E Consorti, R Gonzalez, R Kwan‐Feinberg, T Liu, Z Cooper, S Hirji, D Mahvi, B Okafor, V Roxo, A Salim, A Loehrer, K Telma, M Wilson, M Bokenkamp, AB Haynes, C Hill, E Leede, K McElhinney, KA Olson, C Riley, B Bigelow, EW Etchill, A Gabre‐Kidan, HE Jenny, A Kent, MR Ladd, C Long, H Malapati, A Margalit, S Rapaport, J Rose, K Stevens, L Tsai, D Vervoort, P Yesantharao, D Klaristenfeld, KT Huynh, H Kaafarani, L Naar, M Qadan, DE Cha, E Gleeson, C Horn, U Sarpel, A Bhama, K Colling, M Najarian, M Azam, A Choudhry, W Marx, R Chokshi, N Glass, G Tsui, MK Abel, M Boeck, H Chern, LZ Kornblith, B Nunez‐Garcia, D Ozgediz, A Sarin, MG Varma, D Abbott, A Acher, T Aiken, J Barrett, E Foley, PB Schwartz, SN Zafar, AT Hawkins, A Maiga, NM Ruzgar, M Sion, S Ullrich, H Al‐Naggar, M Al‐Shehari, A Almassaudi, M Alsayadi, R Alsayadi, S Shream

**Keywords:** colorectal cancer, coronavirus, COVID‐19, SARS‐CoV‐2, surgery, surgical delay

## Abstract

**Aim:**

The SARS‐CoV‐2 pandemic has provided a unique opportunity to explore the impact of surgical delays on cancer resectability. This study aimed to compare resectability for colorectal cancer patients undergoing delayed versus non‐delayed surgery.

**Methods:**

This was an international prospective cohort study of consecutive colorectal cancer patients with a decision for curative surgery (January–April 2020). Surgical delay was defined as an operation taking place more than 4 weeks after treatment decision, in a patient who did not receive neoadjuvant therapy. A subgroup analysis explored the effects of delay in elective patients only. The impact of longer delays was explored in a sensitivity analysis. The primary outcome was complete resection, defined as curative resection with an R0 margin.

**Results:**

Overall, 5453 patients from 304 hospitals in 47 countries were included, of whom 6.6% (358/5453) did not receive their planned operation. Of the 4304 operated patients without neoadjuvant therapy, 40.5% (1744/4304) were delayed beyond 4 weeks. Delayed patients were more likely to be older, men, more comorbid, have higher body mass index and have rectal cancer and early stage disease. Delayed patients had higher unadjusted rates of complete resection (93.7% vs. 91.9%, *P* = 0.032) and lower rates of emergency surgery (4.5% vs. 22.5%, *P* < 0.001). After adjustment, delay was not associated with a lower rate of complete resection (OR 1.18, 95% CI 0.90–1.55, *P* = 0.224), which was consistent in elective patients only (OR 0.94, 95% CI 0.69–1.27, *P* = 0.672). Longer delays were not associated with poorer outcomes.

**Conclusion:**

One in 15 colorectal cancer patients did not receive their planned operation during the first wave of COVID‐19. Surgical delay did not appear to compromise resectability, raising the hypothesis that any reduction in long‐term survival attributable to delays is likely to be due to micro‐metastatic disease.

What does this paper add to the literature?

This was a prospective cohort study of 5453 patients with a decision for curative colorectal cancer surgery. Surgical delays of up to 12 weeks were not associated with worse rates of complete resection. Any reduction in long‐term survival attributable to delays is likely to be due to micro‐metastatic disease and should be the focus of postoperative surveillance programmes.

## INTRODUCTION

Globally, colorectal cancer is the third most commonly diagnosed cancer type and the second largest cause of cancer death [1]. The severe acute respiratory syndrome coronavirus 2 (SARS‐CoV‐2) pandemic has affected all aspects of healthcare and has led to variable delays to the delivery of colorectal cancer surgery across the globe [2, 3]. It is estimated that over 28 million operations were cancelled in the initial 3 months of disruption alone [4, 5]. This creates a unique ‘natural experiment’ to explore the effects of treatment delay on outcomes of colorectal cancer surgery.

Although there is no international guidance on the optimal timing for colorectal cancer resection, it is generally perceived as a time critical intervention. In the UK, the National Health Service sets a target of 4 weeks from a treatment decision to definitive treatment in cancer care, but global practice and policy varies significantly. A number of modelling studies and systematic reviews have explored the impact of delays on long‐term survival in colorectal cancer, but it is unclear whether this is related to poorer initial cancer control (i.e., lower rates of complete resection) or micro‐metastatic disease spread [6].

Understanding the effects of surgical delay during the SARS‐CoV‐2 pandemic will help inform future prioritization of surgical waiting lists during post‐pandemic recovery and postoperative surveillance by the multidisciplinary team.

This study aimed to explore the association between delayed surgery for colorectal cancer in patients not undergoing neoadjuvant therapy and surgical resectability during the SARS‐CoV‐2 pandemic.

## METHODS

### Study design and setting

This was an international prospective cohort study which included consecutive patients with a decision for elective curative surgery from the multidisciplinary team meeting, tumour board or equivalent. Any hospital worldwide undertaking elective colorectal cancer surgery was eligible for inclusion in this analysis. Each participating site recruited consecutive eligible patients for a period of 3 months following the emergence of COVID‐19 in their local area (first notification of SARS‐CoV‐2 ranging between January and April 2020). Each site obtained ethical approval according to local regulations, and the COVIDSurg‐Cancer study (overall inclusion by cancer type available in Table S12) was pre‐registered with ClinicalTrials.gov (identifier NCT04384926).

### Patient inclusion, pathways and follow‐up

All patients with a decision for curative cancer surgery or who would normally have been offered curative surgery in the pre‐pandemic setting but an alternative treatment was offered due to COVID‐19 were included. Patients were excluded from this study if they had (1) planned palliative surgery, (2) a suspected cancer that was later found to be benign on histopathology, (3) a suspected benign tumour that was later found to be cancerous or (4) received endoscopic treatment only (e.g., transanal endorectal microsurgery).

From all the included patients, some of them did receive their planned curative surgery but some ended up not receiving it during the study period. For patients who were operated, follow‐up data were collected at 30 days after surgery. For patients who remained non‐operated, their last known status was recorded. All follow‐up was completed by 31 August 2020 with a minimum follow‐up of 3 months for all included patients. The characteristics of non‐operated patients were described and reasons for the non‐performance of the planned surgery were reported. This allows a comprehensive understanding of the whole sample and an informed discussion on how treatment pathways that were in place during the study influenced the patient groups that we are comparing.

Of all the operated patients, some required surgical resection alone and some required neoadjuvant therapy (chemotherapy and/or radiotherapy prior to surgery). Due to differences in disease biology, and potential effects of treatment intervals in patients undergoing neoadjuvant therapy, the patients who received neoadjuvant therapy were excluded from the main analysis as their disease behaviour is expected to be fundamentally different. However, tumour location and the type of neoadjuvant treatment are reported in the supplement for completeness.

### Delay to surgery

The main analysis on surgical delays focused on patients who received their planned surgery with curative intent, without having received neoadjuvant therapy. Delay to surgery was defined according to the number of weeks from the date of the decision for curative surgery to the date when the patient received surgery. For the primary analysis, patients who were operated more than 4 weeks after their decision for surgery were classified as delayed and those who were operated within 4 weeks were defined as non‐delayed. This 4‐week cut‐off was informed by UK National Institute for Clinical Excellence guidance and standards for timely delivery of cancer care [7].

### Outcomes

The primary outcome measure for the study was complete resection, defined as disease amenable to surgical removal at the time of surgery with a negative circumferential resection margin (R0, no microscopic or macroscopic disease within 1 mm of the circumferential resection margin). Patients whose disease became unresectable during the study period or whose surgical resection was achieved with positive resection margins (R1 or R2) were classified as having an incomplete resection.

Secondary outcomes were also compared between patients undergoing delayed and non‐delayed surgery. These included the 30‐day postoperative mortality rate, 30‐day major postoperative complication rate (defined as Clavien–Dindo Grade III–V complications) [8], stage change from baseline (clinical) to pathology (according to the American Joint Committee on Cancer [AJCC] 8th edition of the TNM staging system [9], defined as upstaged for any increase in stage, downstaged for any decrease in stage and no change for patients remaining at the same stage group), detection of new metastatic disease (clinically, intra‐operatively or on radiological imaging that was not present at the time of decision for surgery) and the rate of emergency surgery (i.e., as all patients had an initial plan for elective surgery at study entry, emergency surgery can be interpreted as a cancer‐related complication requiring emergency intervention). The indications for emergency surgery are presented.

### Data variables

Baseline information was collected for each patient at the point of entry to the study. This included age, sex, American Society of Anesthesiologists (ASA) physical status classification, Eastern Cooperative Oncology Group (ECOG) performance score, Revised Cardiac Risk Index (RCRI), body mass index (BMI) (defined as underweight if <18.5 kg/m^2^, normal if 18.5–24.9 kg/m^2^, overweight if 25–29.9 kg/m^2^ or obese if ≥30 kg/m^2^), clinical (based on imaging and clinical observation at the time of decision for surgery) and pathological TNM stage groups collected according to the AJCC 8th edition, country income (grouped as high, upper middle and low/low middle income, as per the World Bank index classification based on gross national income per capita), surgical approach (open, laparoscopic or converted), anastomotic performance (with or without defunctioning stoma) and anastomotic method (handsewn or stapled).

### Data handling and statistics

All the data collected were non‐identifiable and uploaded to a secure online server hosted by the University of Birmingham, using the Research Electronic Data Capture (REDCap) system. Data management and analysis used RStudio version 4.0.3 with the ‘readr’, ‘tidyverse’, ‘dplyr’, ‘gmodels’, ‘Hmisc’ and ‘finalfit’ packages (R Foundation for Statistical Computing). Unadjusted categorical data were compared using the chi‐squared test with Fisher's exact modification where required. A *P* value <0.05 was considered statistically significant. Logistic regression models were used to explore the association between delay to surgery and complete resection, adjusting for clinically plausible patient and disease factors selected a priori. All missing data were recorded and are reported in the tables and figures.

Reflecting differences in treatment timelines and capacity across different resource settings, we performed a sensitivity analysis exploring longer delays of 6, 8 and 12 weeks from treatment decision to surgery, and the primary outcome measure.

Given that patients undergoing emergency surgery could have shown distinct clinical features at the time of decision for surgery that made them more likely to receive an urgent intervention, a pre‐planned subgroup analysis was performed for patients undergoing planned (elective) surgery only. Further subgroup analyses were performed looking exclusively at colon cancers, rectal cancers, early disease and advanced disease. For this purpose, early disease stage was defined as organ confined, non‐nodal, non‐metastatic (T1–3 N0 M0) and advanced disease was defined as reaching the serosa, nodal or metastatic disease (T4, N+ or M1). A sensitivity analysis of the adjusted and unadjusted results was conducted to explore the impact of longer delays in resectability: 0–4, 5–8, 9–12 and more than 12 weeks from decision to surgery.

## RESULTS

In total, 5453 patients eligible for elective curative colorectal cancer surgery were included from 304 hospitals in 47 countries. This corresponds to 24.4% of all the patients included in the COVIDSurg‐Cancer study (the remaining being patients with other cancer types) [10]. Of these 66.3% (3616/5453) had colon cancer and 33.7% (1837/5453) had rectal cancer. The clinical colorectal cancer stage was advanced in 63.6% (3466/5453) of the patients and early in 36.4% (1987/5453). Around two‐thirds of the patients were ASA grade 1–2 (66.7%, 3619/5453) and one‐third were ASA grade 3–5 (33.2%, 1809/5453). The majority of the patients were from high income countries (84.3%, 4599/5453), with 9.2% (500/5453) being from upper middle income countries and 6.4% (351/5453) from lower middle or low income countries.

### Non‐operated patients

**FIGURE 1:**
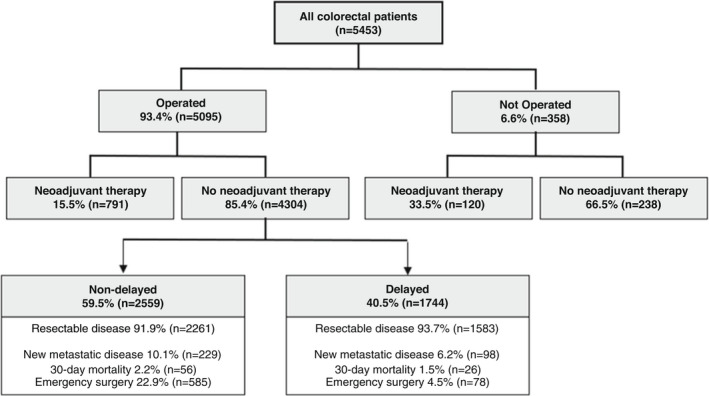
Flowchart of patient inclusion, with outcomes stratified by delay versus non‐delay. Delay was defined as a time from decision to treat to surgery of >4 weeks

From all the included patients, 6.6% (358/5453) did not receive their planned operation during the study period (Figure 1), of whom 74.3% (266/358) were still planned to have curative surgery at the time of follow‐up. Patients who were not operated were more likely to have rectal cancer (52.5% vs. 32.4%, *P* < 0.001), worse performance status (5.9% vs. 2.9% were ECOG 3–4, *P* < 0.001), lower BMI (9.3% vs. 3.5% were underweight, *P* < 0.001), higher stage disease (14.6% vs. 10.3% had clinical Stage IV, *P* = 0.004) and be from a low/lower middle income country (18.2% vs. 5.6%, *P* < 0.001) (Table S1). The reasons why patients did not receive their planned operation are detailed in Table S2, with the most common reasons being a multidisciplinary team decision to avoid surgery due to patient risk (72.6%, 260/358), disease progression (29.1%, 104/358) and patient being unable to travel to hospital during the pandemic (26.3%, 94/358).

### Operated patients

Of the 5095 operated patients, 15.5% (791/5095) received neoadjuvant therapy and 85.4% (4304/5095) underwent surgery without neoadjuvant treatment. The majority of the patients receiving neoadjuvant therapy had rectal cancer (81.8%, 647/791). Neoadjuvant therapy regimens by cancer location are shown in Figure S1.

From the 4304 patients who received an operation without neoadjuvant therapy, 59.5% (2559/4303) had surgery within 4 weeks of treatment decision and 40.5% (1744/4304) were delayed beyond 4 weeks. Delayed patients were more likely to be older (53.0% vs. 46.3% aged over 70 years, *P* < 0.001), men (58.7% vs. 54.6%, *P* = 0.008), more comorbid (37.7% vs. 30.9% were ASA 3–5, *P* < 0.001), have a lower performance status (46.4% vs. 53.4% were ECOG 0), be from a higher income country (90.1% vs. 83.7% were from high income countries), have a higher BMI (22.5% vs. 17.4% were obese) and have a rectal cancer (26.9% vs. 20.8%, *P* < 0.001) and early stage disease (41.9% vs. 32.8% were clinical Stage I). Full demographics are shown in Table 1.

**TABLE 1:** Demographic features of patients having delayed and non‐delayed surgery

		Non‐delayed (*n* = 2559)	Delayed (*n* = 1744)	*P* value
Site	Colon	2028 (79.2)	1274 (73.1)	<0.001
	Rectum	531 (20.8)	470 (26.9)	
Age	<70 years	1374 (53.7)	819 (47.0)	<0.001
	≥70 years	1185 (46.3)	925 (53.0)	
Sex	Female	1162 (45.4)	720 (41.3)	0.008
	Male	1397 (54.6)	1024 (58.7)	
ASA grade	1–2	1764 (69.1)	1084 (62.3)	<0.001
	3–5	789 (30.9)	657 (37.7)	
	Missing	6	3	
ECOG score	0	1343 (53.1)	795 (46.4)	<0.001
	1–2	1101 (43.5)	867 (50.6)	
	3–4	85 (3.4)	50 (2.9)	
	Missing	30	32	
Revised Cardiac Risk Index	1–2	2382 (93.1)	1598 (91.6)	0.086
	≥3	177 (6.9)	146 (8.4)	
Body mass index	Underweight	92 (3.7)	45 (2.6)	<0.001
	Normal	1,121 (44.7)	634 (37.1)	
	Overweight	858 (34.2)	646 (37.8)	
	Obese	437 (17.4)	385 (22.5)	
	Missing	51	34	
Stage group	Stage I	806 (32.8)	709 (41.9)	<0.001
	Stage II	560 (22.8)	365 (21.6)	
	Stage III	863 (35.1)	503 (29.7)	
	Stage IV	230 (9.4)	116 (6.9)	
	Missing	100	51	
Country income	High income	2143 (83.7)	1571 (90.1)	<0.001
	Upper middle income	259 (10.1)	116 (6.7)	
	Low/lower middle income	157 (6.1)	57 (3.3)	
Approach	Open	1203 (47.1)	800 (45.9)	0.733
	Minimally invasive	1216 (47.6)	850 (48.8)	
	Converted to open	137 (5.4)	92 (5.3)	
	Missing	3	2	
Anastomosis	Yes (with defunctioning stoma)	330 (13.1)	199 (11.6)	0.316
	Yes (without defunctioning stoma)	1716 (68.3)	1187 (69.1)	
	No	467 (18.6)	331 (19.3)	
	Missing	46	27	
Anastomotic method	Stapled	1646 (80.5)	1125 (81.2)	0.641
	Handsewn	398 (19.5)	260 (18.8)	
	Missing	515	359	

*Notes*: Delay was defined as a time from decision to treat to surgery of >4 weeks. Data reported as *n* (%). Percentages expressed of column total. *P* values calculated using chi‐squared test.

Abbreviations: ASA, American Society of Anesthesiologists classification; ECOG, Eastern Cooperative Oncology Group.

### Outcomes of delayed surgery

**FIGURE 2:**
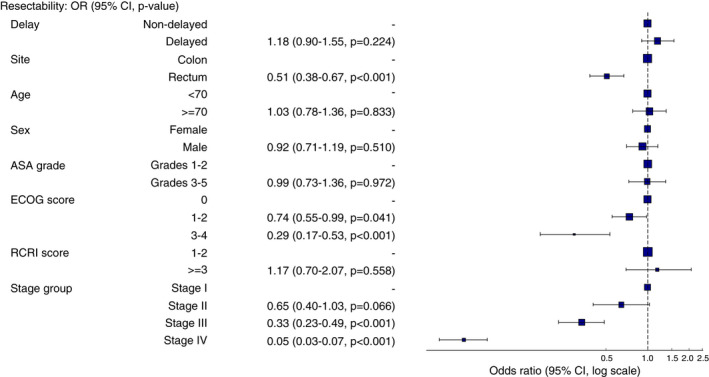
Multivariate logistic regression model exploring the association between delay to surgery and resectability, adjusting for patient and disease factors. Number in dataframe 3966, number in model 3966, missing 0, Akaike information criterion 1786.9, *C* statistic 0.776. Full model presented in Table S4. Delay was defined as a time from decision to treat to surgery of >4 weeks. Data reported as odds ratio (95% confidence interval, *P* value). OR >1 means higher odds of resectability for delayed patients, OR <1 means lower odds of resectability for delayed patients. ASA, American Society of Anesthesiologists classification; ECOG, Eastern Cooperative Oncology Group; RCRI. Revised Cardiac Risk Index

Delayed patients did not have lower rates of complete resection, compared to non‐delayed patients. In the unadjusted analysis, delayed patients were more likely to have resectable disease (93.7% vs. 91.9%, *P* = 0.032) and less likely to develop new metastases (6.2% vs. 10.1%, *P* < 0.001) than non‐delayed patients. Changes in disease stage from baseline to pathological staging were more common in delayed patients, including both upstaging and downstaging (Table 2). Delayed patients were also less likely to have had emergency surgery (4.5% vs. 22.9%, *P* < 0.001) whilst waiting for their planned surgery, mainly due to obstructive symptoms. Other indications for emergency surgery in this cohort are shown in Table S3. There were no significant differences in 30‐day major postoperative complications (9.3% vs. 9.8%, *P* = 0.648) or postoperative mortality rates (1.5% vs. 2.2%, *P* = 0.126). After adjustment for case mix, delay was not associated with significantly lower rates of complete resection (OR = 1.18, 95% CI 0.90–1.55, *P* = 0.224) (Figure 2). The full adjusted model can be found in Table S4.

**TABLE 2:** Unadjusted outcomes compared between delayed and non‐delayed patients

		Non‐delayed (*n* = 2559)	Delayed (*n* = 1744)	*P* value
Resectability	Complete resection	2261 (91.9)	1583 (93.7)	0.032
	Incomplete resection	199 (8.1)	106 (6.3)	
	Missing	99	55	
Resection margins	Positive	107 (4.4)	74 (4.4)	1
	Negative	2310 (95.6)	1599 (95.6)	
	Missing	142	71	
Progression to unresectable disease	Yes	127 (5.0)	40 (2.3)	<0.001
	No	2432 (95.0)	1703 (97.7)	
	Missing	0	1	
New metastatic disease	Yes	229 (10.1)	98 (6.2)	<0.001
	No	2036 (89.9)	1472 (93.8)	
	Missing	294	174	
Stage change (from baseline to pathology)	Downstaged	393 (18.1)	335 (22.0)	0.001
	No change	1236 (56.9)	775 (50.8)	
	Upstaged	543 (25.0)	416 (27.3)	
	Missing	387	218	
30‐day mortality	Died	56 (2.2)	26 (1.5)	0.126
	Alive	2502 (97.8)	1718 (98.5)	
	Missing	1	0	
30‐day major postoperative complications	Yes	251 (9.8)	163 (9.3)	0.648
	No	2307 (90.2)	1581 (90.7)	
	Missing	1	0	
Urgency	Emergency	585 (22.9)	78 (4.5)	<0.001
	Elective	1973 (77.1)	1663 (95.5)	
	Missing	1	3	

*Notes*: Delay was defined as a time from decision to treat to surgery of >4 weeks. Data reported as *n* (%). Percentages expressed of column total. *P* values calculated using chi‐squared test.

### Subgroup analysis

In the subgroup analysis of patients undergoing elective surgery only, delay was not associated with lower rates of complete resection (OR = 0.94, 95% CI 0.69–1.27, *P* = 0.672) (logistic regression model available in Figure S2). Demographic trends of delayed patients were also similar to the main analysis (Table S5). When looking at colon and rectal cancers in isolation, a delay of 4 weeks was not associated with a reduced rate of complete resection in colon (OR = 1.33, 95% CI 0.95–1.87, *P* = 0.101) or rectal cancer (OR = 0.91, 95% CI 0.58–1.44, *P* = 0.692). Delay was not associated with poorer resectability in patients with early disease only (OR = 1.20, 95% CI 0.67–2.18, *P* = 0.537) or advanced disease only (OR = 1.11, 95% CI 0.81–1.52, *P* = 0.517). Full logistic regression models for the subgroup analysis are shown in Tables S6–S9.

### Sensitivity analysis of longer surgical delays

In a sensitivity analysis exploring the association of longer delays and complete resection, 59.5% (2559/4304) of patients were operated in 0–4 weeks, 25.3% (1089/4304) in 5–8 weeks, 8.9% (384/4304) in 9–12 weeks and 6.3% (271/4304) in >12 weeks from decision to surgery (all demographics available in Table S10). Longer delays were not associated with worse resectability outcomes in unadjusted (Table S11) or adjusted analyses (Table 3). Compared to patients undergoing surgery within 4 weeks of treatment decision, the odds of complete resection were not significantly different at 5–8 weeks from treatment decision (OR = 1.16, 95% CI 0.86–1.59, *P* = 0.344), at 9–12 weeks (OR = 1.40, 95% CI 0.85–2.41, *P* = 0.206) or beyond 12 weeks (OR = 1.03, 95% CI 0.62–1.80, *P* = 0.920).

**TABLE 3:** Multivariate logistic regression model exploring the association between stratified delay to surgery and resectability, adjusting for patient and disease factors

		Non‐resectable (*n* = 297)	Resectable (*n* = 3669)	OR (univariable)	OR (multivariable)
Delay	0–4 weeks	193 (8.2)	2154 (91.8)	–	–
	5–8 weeks	66 (6.5)	955 (93.5)	1.30 (0.98–1.74, *P* = 0.079)	1.16 (0.86–1.59, *P* = 0.344)
	9–12 weeks	19 (5.3)	338 (94.7)	1.59 (1.01–2.67, *P* = 0.060)	1.40 (0.85–2.41, *P* = 0.206)
	>12 weeks	19 (7.9)	222 (92.1)	1.05 (0.66–1.76, *P* = 0.855)	1.03 (0.62–1.80, *P* = 0.920)
Site	Colon	200 (6.6)	2846 (93.4)	–	–
	Rectum	97 (10.5)	823 (89.5)	0.60 (0.46–0.77, *P* < 0.001)	0.51 (0.38–0.68, *P* < 0.001)
Age	<70	158 (7.9)	1850 (92.1)	–	–
	≥70	139 (7.1)	1819 (92.9)	1.12 (0.88–1.42, *P* = 0.358)	1.03 (0.78–1.36, *P* = 0.828)
Sex	Female	127 (7.3)	1604 (92.7)	–	–
	Male	170 (7.6)	2065 (92.4)	0.96 (0.76–1.22, *P* = 0.749)	0.91 (0.70–1.18, *P* = 0.491)
ASA grade	1–2	187 (7.1)	2437 (92.9)	–	–
	3–5	110 (8.2)	1232 (91.8)	0.86 (0.67–1.10, *P* = 0.226)	0.99 (0.73–1.36, *P* = 0.964)
ECOG grade	0	129 (6.4)	1874 (93.6)	–	–
	1–2	144 (7.9)	1690 (92.1)	0.81 (0.63–1.03, *P* = 0.090)	0.74 (0.55–0.98, *P* = 0.039)
	3–4	24 (18.6)	105 (81.4)	0.30 (0.19–0.50, *P* < 0.001)	0.29 (0.17–0.53, *P* < 0.001)
RCRI grade	1–2	278 (7.6)	3387 (92.4)	–	–
	≥3	19 (6.3)	282 (93.7)	1.22 (0.77–2.03, *P* = 0.421)	1.18 (0.71–2.08, *P* = 0.544)
Stage group	Stage I	39 (2.7)	1418 (97.3)	–	–
	Stage II	35 (4.0)	838 (96.0)	0.66 (0.41–1.05, *P* = 0.078)	0.65 (0.40–1.03, *P* = 0.066)
	Stage III	102 (7.8)	1205 (92.2)	0.32 (0.22–0.47, *P* < 0.001)	0.34 (0.23–0.49, *P* < 0.001)
	Stage IV	121 (36.8)	208 (63.2)	0.05 (0.03–0.07, *P* < 0.001)	0.05 (0.03–0.07, *P* < 0.001)

*Notes*: Number in dataframe 3966, number in model 3966, missing 0, Akaike information criterion 1790.1, *C* statistic 0.776. Delay was measured from decision to treat to surgery. Data reported as odds ratio (95% confidence interval, *P* value). OR >1 means higher odds of resectability for delayed patients, OR <1 means lower odds of resectability for delayed patients.

Abbreviations: ASA, American Society of Anesthesiologists classification; ECOG, Eastern Cooperative Oncology Group; RCRI, Revised Cardiac Risk Index.

## DISCUSSION AND CONCLUSIONS

During the first wave of the SARS‐CoV‐2 pandemic, one in 15 patients did not receive their planned operation for colorectal cancer. In those who did undergo surgery, delays of more than 4 weeks did not appear to be associated with reduced rates of complete resection. This was robust to several sensitivity and subgroup analyses. Although there are inherent biases in this study design, including selection bias in those that were exposed to treatment delay, this study represents a unique natural experiment to better understand the pathobiology of survival after colorectal cancer surgery.

Whilst long‐term oncological outcome data are not yet available for this cohort, these data provide important insight into the potential mechanism for the relationship between long‐term survival and treatment delay. Although the previous studies show controversial findings on the impact of delay to oncological outcomes [11, 12, 13, 14], a systematic review looking at long‐term survival for patients undergoing colorectal cancer surgery 1 month and 3 months after the diagnosis showed a reduction in overall and disease‐free survival with surgical delays [15]. Another multi‐specialty review of delays in multimodal cancer treatment showed a negative impact on long‐term oncological outcomes [6]. This study suggests that a delay to surgery does not affect short‐term patho‐oncological outcomes. It raises the hypothesis that any decrease in long‐term survival observed is unlikely to be due to initial cancer control and may be related to micro‐metastatic disease spread. Patients whose surgery is delayed might therefore benefit from closer follow‐up strategies for early detection of relapse and metastatic disease. Further research is required to understand the effectiveness of enhanced follow‐up pathways on long‐term survival, alongside their performance in different tumour biology patterns (not captured in this study).

The clinical features of non‐operated patients suggest clinical selection based on a perceived high risk of surgical complications, given that these patients had worse performance status, were more likely to be underweight and had more advanced disease. Although these decisions probably aimed to protect frail patients from the additional risk conveyed by perioperative SARS‐CoV‐2 infection, they might have exposed some patients with advanced disease to a risk of progression to palliative disease. Changes in the management of colorectal cancer during the COVID‐19 pandemic have been described by several research groups, including reduction of the number of patients receiving surgery and shorter treatment regimens [16, 17, 18]. This study provides further insight on the drivers of these clinical decisions and on which patients might have been more impacted by them.

Advanced (non‐organ confined) and rectal cancers were also more likely to be operated promptly, as opposed to early and colon cancers which were more likely to be delayed. This suggests that additional features of the disease were perceived by surgical teams as justifying early surgery, which might explain why non‐delayed patients had higher non‐adjusted rates of progression to unresectable disease and new metastasis. Changes in disease stage observed in this study include higher rates of both upstage and downstage with increased delay. As delayed patients were more likely to have advanced disease, this might reflect lower reliability of clinical staging and imaging studies in advanced cancers, particularly when nodal disease is present [19].

The performance of elective colorectal cancer surgery within 4 weeks of treatment decision might not be feasible in many settings worldwide, even in a pre‐pandemic setting [20, 21]. Additionally, there might be variation in the usual timeframes from decision to surgery across settings, depending on local practices and pathways (e.g., preoperative assessment efficiency, existence of routine pre‐habilitation programmes). This study looked at longer delays of 8 and 12 weeks which showed no association with resectability impairment either, ensuring the generalizability of the findings.

Symptoms of obstruction, perforation or bleeding in patients awaiting elective surgery might have prompted earlier surgery, explaining why emergency surgery was more common in non‐delayed patients (undergoing surgery within 4 weeks of treatment decision). Although we presented the reasons for emergency surgery in this cohort of patients awaiting planned resection, some could have had symptoms of obstruction or other acute complication at the time of treatment decision, to whom a delay beyond 4 weeks would not be clinically acceptable. To address the selection bias that these clinical findings might have had in the length of delay from decision to surgery, we performed a subgroup analysis of patients undergoing elective surgery only, that again showed no difference in resectability with surgical delays.

This study has several important limitations. Longer‐term follow‐up of this cohort will be required to explore the true clinical impact of treatment delay for these patients. The second is the risk of selection bias in the comparison of delayed and non‐delayed patients. We attempted to overcome this through multivariable modelling and several subgroup and sensitivity analyses, but the analysis may still be subject to residual bias from unmeasured confounders. Third, patients who remained non‐operated may have had a poorer prognosis at baseline and/or may have been subject to disease progression and other cancer‐related sequelae which could lead to underestimation of the impact of delay (7% of the cohort overall). Fourth, we were unable to explore the impact of treatment delay in patients with prior neoadjuvant therapy, who pose a biologically distinct treatment group. Finally, histological data were not collected and therefore we were unable to explore whether molecular subtypes or mutational status differed between the groups, and whether this impacted resection.

SARS‐CoV‐2 waves are not the only pressure that health systems face, and many factors can cause delays in the delivery of surgical care. These findings can inform clinical decision making, management of surgical waiting lists and patient informed consent before surgery. Guidance on management of colorectal cancer should also take these findings into account when designing follow‐up strategies for patients who are operated for colorectal cancer. The possibility of performing cancer resection with a few weeks of delay without a negative impact on local control could be important for patients who may benefit from longer periods of pre‐habilitation and pre‐conditioning before surgery, in order to achieve a better fitness status and optimize perioperative outcomes.

## Funding information

This study was funded and supported by the National Institute for Health Research Global Health Research Unit on Global Surgery, Association of Coloproctology of Great Britain and Ireland, Bowel and Cancer Research, Bowel Disease Research Foundation, Association of Upper Gastrointestinal Surgeons, British Association of Surgical Oncology, British Gynaecological Cancer Society, European Society of Coloproctology, Medtronic, Sarcoma UK, the Urology Foundation, Vascular Society for Great Britain and Ireland, and Yorkshire Cancer Research. The funders did not interfere with the data collection or presentation of results.

## AUTHOR CONTRIBUTION

Individual contributions to this paper are listed in the appendix. The writing group and statistical analysis group have analysed, interpreted and drawn conclusions from the data. The COVIDSurg operations team, international cancer leads and dissemination committees led the conduct of the study and contributed to data curation. The listed collaborators have contributed with patient level data from their sites.

## CONFLICT OF INTEREST

There are no conflicts of interest to declare.

## ETHICAL STATEMENT

This study was approved in every participating country and hospital as per local requirements. National and hospital leads were responsible and guaranteed the necessary approvals ahead of data upload.

## Supporting information


Appendix S1

## Data Availability

Data‐sharing requests will be considered by the management group upon written request to the corresponding author. If agreed, de‐identified participant data will be available, subject to a data‐sharing agreement.

## APPENDIX A

### List of PubMed citable COVIDSurg collaborators

*Writing group*: Michel Adamina (Switzerland), Adesoji Ademuyiwa (Nigeria), Adewale Adisa (Nigeria), Aneel A Bhangu (UK), Ana Minaya Bravo (Spain), Miguel F Cunha (Portugal), Sameh Emile (Egypt), Dhruva Ghosh (India), James C Glasbey (UK), Benjamin Harris (UK)*, Debby Keller (USA), Samuel Lawday (UK), Hans Lederhuber (Germany), Sezai Leventoglu (Turkey), Elizabeth Li (UK), Maria Marta Modolo (Argentina), Rohin Mittal (India), Helen M Mohan (Ireland), Dmitri Nepogodiev (UK), Marie Dione Parreño‐Sacdalan (Philippines), Francesco Pata (Italy), Peter Pockney (Australia), Martin Rutegård (Sweden), Joana FF Simões (Portugal)*, Neil Smart (UK), Chris Varghese (New Zealand).

*Joint first authors.

*Statistical analysis and data handling*

James C Glasbey, Joana FF Simoes, Benjamin Harris, Aneel A Bhangu.

*COVIDSurg Operations Committee*

Dmitri Nepogodiev (***Chair***), Kwabena Siaw‐Acheampong, Ruth A Benson, Edward Bywater, Daoud Chaudhry, Brett E Dawson, Jonathan P Evans, James C Glasbey, Rohan R Gujjuri, Emily Heritage, Conor S Jones, Sivesh K Kamarajah, Chetan Khatri, Rachel A Khaw, James M Keatley, Andrew Knight, Samuel Lawday, Elizabeth Li, Harvinder S Mann, Ella J Marson, Kenneth A McLean, Siobhan C Mckay, Emily C Mills, Gianluca Pellino, Maria Picciochi, Elliott H Taylor, Abhinav Tiwari, Joana FF Simoes, Isobel M Trout, Mary L Venn, Richard JW Wilkin, Aneel Bhangu.

*International Cancer Leads (*denotes specialty Principal Investigators)*

James C Glasbey (***Chair***)

**Colorectal:** Neil J Smart*, Ana Minaya‐Bravo*, Jonathan P Evans, Gaetano Gallo, Susan Moug, Francesco Pata, Peter Pockney, Salomone Di Saverio, Abigail Vallance, Dale Vimalchandran.

**Oesophagogastric**: Ewen A Griffiths*, Sivesh K Kamarajah, Richard PT Evans, Philip Townend.

**Hepatopancreatobiliary**: Keith Roberts*, Siobhan McKay*, John Isaac, Sohei Satoi.

**Thoracic:** John Edwards*, Aman S Coonar, Adrian Marchbank, Edward J Caruana, Georgia R Layton, Akshay Patel, Alessandro Brunelli.

**Sarcoma:** Samuel Ford*, Anant Desai*, Alessandro Gronchi*, Marco Fiore*, Max Almond, Fabio Tirotta, Sinziana Dumitra.

**Neurosurgery:** Angelos Kolias*, Stephen J Price, Daniel M Fountain, Michael D Jenkinson, Peter Hutchinson, Hani J Marcus, Rory J Piper, Laura Lippa, Franco Servadei, Ignatius Esene, Christian Freyschlag, Iuri Neville, Gail Rosseau, Karl Schaller, Andreas K Demetriades, Faith Robertson, Alex Alamri.

**Head and neck:** Richard Shaw*, Andrew G Schache, Stuart C Winter, Michael Ho, Paul Nankivell, Juan Rey Biel, Martin Batstone, Ian Ganly.

**Breast:** Raghavan Vidya*, Alex Wilkins, Jagdeep K Singh, Dinesh Thekinkattil.

**Gynaecology:** Sudha Sundar*, Christina Fotopoulou*, Elaine YL Leung, Tabassum Khan, Luis Chiva, Jalid Sehouli, Anna Fagotti, Paul Cohen, Murat Gutelkin, Rahel Ghebre, Thomas Konney, Rene Pareja, Rob Bristow, Sean Dowdy, Shylasree TS, Rajkumar Kottayasamy Seenivasagam, Joe Ng, Keiichi Fujiwara.

**Urology:** Grant D Stewart*, Benjamin Lamb, Krishna Narahari, Alan McNeill, Alexandra Colquhoun, John S McGrath, Steve Bromage, Ravi Barod, Veeru Kasivisvanathan*, Tobias Klatte.

*Dissemination Committee*

Joana FF Simoes (***Chair***); Tom EF Abbott, Sadi Abukhalaf, Michel Adamina, Adesoji O Ademuyiwa, Arnav Agarwal, Murat Akkulak, Ehab Alameer, Derek Alderson, Felix Alakaloko, Markus Albertsmeier, Osaid Alser, Muhammad Alshaar, Sattar Alshryda, Alexis P Arnaud, Knut Magne Augestad, Faris Ayasra, José Azevedo, Brittany K Bankhead‐Kendall, Emma Barlow, David Beard, Ruth A Benson, Ruth Blanco‐Colino, Amanpreet Brar, Ana Minaya‐Bravo, Kerry A Breen, Chris Bretherton, Igor Lima Buarque, Joshua Burke, Edward J Caruana, Mohammad Chaar, Sohini Chakrabortee, Peter Christensen, Daniel Cox, Moises Cukier, Miguel F Cunha, Giana H Davidson, Anant Desai, Salomone Di Saverio, Thomas M Drake, John G Edwards, Muhammed Elhadi, Sameh Emile, Shebani Farik, Marco Fiore, J Edward Fitzgerald, Samuel Ford, Tatiana Garmanova, Gaetano Gallo, Dhruva Ghosh, Gustavo Mendonça Ataíde Gomes, Gustavo Grecinos, Ewen A Griffiths, Magdalena Gruendl, Constantine Halkias, Ewen M Harrison, Intisar Hisham, Peter J Hutchinson, Shelley Hwang, Arda Isik, Michael D Jenkinson, Pascal Jonker, Haytham MA Kaafarani, Debby Keller, Angelos Kolias, Schelto Kruijff, Ismail Lawani, Hans Lederhuber, Sezai Leventoglu, Andrey Litvin, Andrew Loehrer, Markus W Löffler, Maria Aguilera Lorena, Maria Marta Modolo, Piotr Major, Janet Martin, Hassan N Mashbari, Dennis Mazingi, Symeon Metallidis, Ana Minaya‐Bravo, Helen M Mohan, Rachel Moore, David Moszkowicz, Susan Moug, Joshua S Ng‐Kamstra, Mayaba Maimbo, Ionut Negoi, Milagros Niquen, Faustin Ntirenganya, Maricarmen Olivos, Kacimi Oussama, Oumaima Outani, Marie Dione Parreño‐Sacdalan, Francesco Pata, Carlos Jose Perez Rivera, Thomas D Pinkney, Willemijn van der Plas, Peter Pockney, Ahmad Qureshi, Dejan Radenkovic, Antonio Ramos‐De la Medina, Toby Richards, Keith Roberts, April C Roslani, Martin Rutegård, Juan José Segura‐Sampedro, Irène Santos, Sohei Satoi, Raza Sayyed, Andrew Schache, Andreas A Schnitzbauer, Justina O. Seyi‐Olajide, Neil Sharma, Catherine A Shaw, Richard Shaw, Sebastian Shu, Kjetil Soreide, Antonino Spinelli, Grant D Stewart, Malin Sund, Sudha Sundar, Stephen Tabiri, Philip Townend, Georgios Tsoulfas, Gabrielle H van Ramshorst, Raghavan Vidya, Dale Vimalachandran, Oliver J Warren, Duane Wedderburn, Naomi Wright, EuroSurg, European Society of Coloproctology (ESCP), Global Initiative for Children’s Surgery (GICS), GlobalSurg, GlobalPaedSurg, ItSURG, PTSurg, SpainSurg, Italian Society of Colorectal Surgery (SICCR), Association of Surgeons in Training (ASiT), Irish Surgical Research Collaborative (ISRC), Transatlantic Australasian Retroperitoneal Sarcoma Working Group (TARPSWG), Italian Society of Surgical Oncology (SICO).

*Collaborators (*denotes hospital leads)*

Argentina: Valenzuela JI* (Hospital Velez Sarsfield, City of Buenos Aires); Alurralde C, Caram EL, Eskinazi DG* (Sanatorio 9 De Julio Sa, Tucuman); Badra R, García JS, Lucchini SM* (Sanatorio Allende, Cordoba).

Australia: Vasey C*, Watson E (Ballarat Base Hospital, Ballarat Central); Cecire J, Salindera S*, Sutherland A (Coffs Harbour Health Campus, Coffs Harbour NSW); Ahn JH, Chen S, Gauri N, Jang S, Jia F, Mulligan CS, Yang W, Ye G, Zhang H (Concord Repatriation General Hospital, Concord West, Sydney); Moss J*, Richards T, Thian A, Vo UG* (Fiona Stanley Hospital, Perth); Bagraith K, Chan E, Ho D, Jeyarajan E, Jordan S, Nolan GJ, Von Papen M, Wullschleger M (Gold Coast University Hospital, Southport); Dawson AC*, Drane A (Gosford Hospital, Gosford); Egoroff N, Gani J, Lott N, Pockney P* (John Hunter Hospital, Newcastle); Phan D, Townend D* (Lismore Base Hospital, Lismore); Bong C, Gundara J* (Logan Hospital, Brisbane); Bowman S*, Guerra GR (Queen Elizabeth II Jubilee Hospital, Brisbane); Gerns N, Riddell A* (Redcliffe Hospital, Redcliffe); Dudi‐Venkata NN, Kroon HM*, Sammour T (Royal Adelaide Hospital, Adelaide); Mitchell D*, Swinson B (Royal Brisbane and Women’s Hospital, Brisbane); Waldron A, Walker P* (St John of God Midland Public and Private Hospital, Perth); Dawson AC*, Drane A*, Lun EWY* (Wyong Public Hospital, Wyong).

Austria: Messner F, Öfner D* (Medical University of Innsbruck, Innsbruck); Emmanuel K, Grechenig M, Gruber R, Harald M, Jäger T, Öhlberger L, Presl J*, Wimmer A (Paracelsus Medical University Salzburg, Salzburg).

Azerbaijan: Namazov İ, Samadov E (Leyla Medical Centerl, Baku).

Barbados: Barker D, Boyce R, Doyle A, Eastmond A, Gill R, O’Shea M, Padmore G*, Paquette N, Phillips E, St John S, Walkes K (Queen Elizabeth Hospital, Bridgetown).

Belgium: Flamey N, Pattyn P* (Az Delta, Roeselare); Ceelen W, Pattyn P, Van de Putte D, Van Nieuwenhove Y, Van Ramshorst G*, Willaert W (University Hospital of Ghent, Gent); Oosterlinck W*, Van den Eynde J, Van den Eynde R (Uz Leuven, Leuven).

Brazil: Aguiar Júnior S*, Marques T (A.c. Camargo Cancer Center, São Paulo); Camara P*, De Lima RK, Della Giustina E, Hoffmann PV (Fundação Hospitalar De Blumenau, Blumenau); Nacif L* (Hospital Nove De Julho, Sao Paulo); Carvalho Ferro C, Gomes GMA, Lima Buarque I, Lira dos Santos Leite A, Pol‐Fachin L, Santos Bezerra T, Silva A, Silvestre D, Vieira Barros A* (Hospital Santa Casa De Misericordia De Maceio, Maceio); Laporte G*, Salem M (Irmandade Da Santa Casa De Misericórdia De Porto Alegre, Porto Alegre); Barakat Awada J, Ijichi TR, Kim NJ, Marreiro A, Muller B, Nunes R* (Notre Dame Intermédica—Hospital Salvalus, São Paulo); Bodanese B, Isoton JC, Regina de Sampaio L, Vendrame C* (Supera Oncologia—Hospital Regional Do Oeste, Chapeco).

Bulgaria: Sokolov M*, Gribnev P (University Hospital Alexandrovska, Sofia).

Canada: Boutros M*, Caminsky N, Ghitulescu G (Jewish General Hospital, Montreal); Groot G*, Persad A, Pham H, Wood M (Saskatoon City Hospital/Royal University Hospital/St Paul’s Hospital, Saskatoon Sk); Boutros M*, Demyttenaere S*, Garfinkle R (St Mary’s Hospital, Montreal); Brown C*, Karimuddin A, Lee N, Liu J, Madani Kia T, Phang PT, Raval M, Tom K (St Paul’s Hospital, Vancouver, BC); Martel A, Nessim C*, Stevenson J (The Ottawa Hospital, Ottawa); Al Riyami S, Bali K, Bigam D*, Dajani K, Dell A (University of Alberta Hospital, Edmonton).

Chile: Bellolio F*, Besser N, Grasset E*, Inzunza M, Quintana Martinic M, Riquoir Altamirano C, Ruiz Esquide M (Hospital Clinico Universidad Católica, Santiago).

Colombia: Arias‐Amézquita F*, Cétares C, Cortes Murgueitio N, Gomez‐Mayorga JL (Fundacion Santa Fe De Bogota, Bogota); Abadia M, Bonilla A, Facundo H, Guevara O*, Herrera Mora DR, Jimenez Ramirez LJ, Manrique E, Pinilla Morales RE*, Rey Ferro M, Velasquez Cuasquen BG (Instituto Nacional De Cancerologia, Bogota).

Croatia: Bačić G, Karlović D, Kršul D, Zelić M* (University Hospital Center Rijeka, Rijeka); Bakmaz B, Ćoza I, Dijan E, Katusic Z, Mihanovic J*, Rakvin I (Zadar General Hospital, Zadar).

Cyprus: Almezghwi H, Arslan K, Özant A, Özçay N* (Near East University Hospital, Nicosia); Frantzeskou K, Gouvas N*, Kokkinos G, Papatheodorou P, Pozotou I, Stavrinidou O, Yiallourou A* (Nicosia General Hospital, Nicosia).

Czechia: Martinek L, Skrovina M*, Szubota I (Hospital and Oncological Centre Novy Jicin, Novy Jicin); Peteja M, Žatecký J* (Slezská Nemocnice V Opavě, P.o., Opava).

Denmark: Avlund T, Christensen P*, Harbjerg JL, Iversen LH, Kjaer DW, Kristensen HØ, Mekhael M (Aarhus University Hospital, Aarhus); Ebbehøj AL, Krarup P, Schlesinger N, Smith H* (Bispebjerg Hospital, Copenhagen).

Dominican Republic: Crespo A, Díaz P, Rivas R, Tactuk N (Cedimat—Centro De Diagnóstico, Medicina Avanzada, Laboratorio Y Telemedicina, Santo Domingo).

Egypt: El Kassas M*, Omar W, Tawheed A (15th May Hospital—Helwan University, Cairo); Talaat M (Ain Shams University Specialized Hospital, Cairo); Abdelsamed A, Azzam AY*, Salem H*, Seleim A (Al Azhar University Hospitals, Cairo); AL Sayed M, Ashoush F*, Elazzazy E, Essam E, Ewedah M, Hassan E, Metwalli M, Mourad M, Qatora MS, Sabry A*, Samih A, Samir Abdelaal A, Shehata S*, Shenit K (Alexandria Main University Hospital, Alexandria); Attia D, Kamal N, Osman N* (Alexandria Medical Research Institute, Alexandria); Alaa S, Hamza HM, M.elghazaly S, Mohammed MM*, Nageh MA, Saad MM*, Yousof EA (Assiut University Hospital, Assiut); Eldaly A* (El‐Menshawy Hospital, Tanta); Alrahawy M*, Sakr A*, Soliman H*, Soltan H* (Menofiya University Hospital, Menoufia); Amira G, Sallam I*, Sherief M, Sherif A (Misr Cancer Center, Al Jizah); Ghaly G*, Hamdy R, Morsi A, Salem H*, Sherif G (National Cancer Institute, Cairo); Abdeldayem H, Abdelkader Salama I*, Balabel M, Fayed Y, Ahmed Elshawadfy Sherif* (National Liver Institute, Menoufia University, Shibin Elkom); Elmorsi R*, Refky B* (Oncology Center Mansoura University, Mansoura).

Ethiopia: Bekele K* (Maddawalabu University Goba Referral Hospital, Goba).

Finland: Kauppila JH*, Sarjanoja E (Länsi‐Pohja Central Hospital, Kemi); Helminen O, Huhta H, Kauppila JH* (Oulu University Hospital, Oulu).

France: Beyrne C, Jouffret L*, Marie‐Macron L (Centre Hospitalier Avignon, Avignon); Fredon F*, Roux A (Centre Hospitalier Roland Mazoin, Saint‐Junien); Lakkis Z*, Manfredelli S (University Hospital of Besançon, Besancon); Chebaro A*, El Amrani M, Lecolle K, Piessen G*, Eveno C, Noiret B, Veziant J Pruvot FR, Zerbib P (Chu Lille, Lille); Ballouhey Q*, Barrat B, Taibi A (Chu Limoges, Limoges); Bergeat D, Merdrignac A (Chu Rennes—General Surgery, Rennes); Le Roy B, Perotto LO, Scalabre A* (Chu Saint Etienne, Saint Etienne); Aimé A, Ezanno AC*, Malgras B (Hia Begin, St Mande); Bouché PA*, Tzedakis S* (Hôpital Cochin—Aphp, Paris); Cotte E, Glehen O (Hopital Lyon Sud, Pierre Bénite); Bendjemar L, Braham H, Charre L, El Arbi N, Police A*, Volpin E (Hôpital Simone Veil, Eaubonne); D’Urso A, Mutter D, Seeliger B* (Strasbourg University Hospitals/IHU‐Strasbourg, Strasbourg); Bonnet S, Denet C, Fuks D, Laforest A, Pourcher G, Seguin‐givelet A*, Tribillon E (Institut Mutualiste Montsouris, Paris); Duchalais E* (Nantes University Hospital, Nantes).

Germany: Bork U*, Fritzmann J, Praetorius C, Weitz J, Welsch T ( University Hospital Carl Gustav Carus, TU Dresden, Dresden); Beyer K, Kamphues C*, Lauscher J, Loch FN, Schineis C (Charité University Medicine, Campus Benjamin Franklin, Berlin); Albertsmeier M*, Kappenberger A, Schiergens T, Werner J (Department of General, Visceral and Transplantation Surgery, LMU University Hospital, Ludwig‐Maximilians‐Universität Munich, Munich); Becker R*, Jonescheit J (Heilig‐Geist Hospital Bensheim, Bensheim); Pergolini I, Reim D* (Klinikum Rechts Der Isar, Tum School of Medicine, Munich); Herzberg J*, Honarpisheh H*, Strate T* (Krankenhaus Reinbek St Adolf‐Stift, Reinbek); Boeker C, Hakami I*, Mall JW* (KRH Nordstadt‐Siloah Hospitals, Hannover); Nowak K*, Reinhard T* (Romed Klinikum Rosenheim, Rosenheim); Kleeff J, Michalski C, Ronellenfitsch U* (University Hospital Halle (Saale), Halle (Saale)); Bertolani E, Königsrainer A*, Löffler MW, Quante M*, Steidle C, Überrück L, Yurttas C (University Hospital Tübingen, Tübingen); Izbicki J, Nitschke C, Perez D, Uzunoglu FG* (University Medical Center Hamburg‐Eppendorf, Hamburg).

Greece: Antonakis P, Contis I, Dellaportas D, Gklavas A, Konstadoulakis M, Memos N*, Papaconstantinou I*, Polydorou A, Theodosopoulos T, Vezakis A (Aretaieion Hospital, Athens); Antonopoulou MI, Manatakis DK*, Tasis N (Athens Naval and Veterans Hospital, Athens); Arkadopoulos N, Danias N, Economopoulou P, Frountzas M, Kokoropoulos P, Larentzakis A, Michalopoulos N*, Parasyris S, Selmani J, Sidiropoulos T, Vassiliu P (Attikon University General Hospital, Athens); Bouchagier K*, Klimopoulos S, Paspaliari D, Stylianidis G (Evaggelismos General Hospital, Athens); Akrivou D, Baxevanidou K, Bouliaris K, Chatzikomnitsa P, Delinasios G, Doudakmanis C, Efthimiou M, Giaglaras A, Kalfountzos C*, Kolla C, Koukoulis G, Zervas K, Zourntou S (General Hospital of Larissa ‘Koutlimpaneio and Triantafylleio’, Larissa); Baloyiannis I, Diamantis A, Perivoliotis K, Tzovaras G* (General University Hospital of Larissa, Larrisa); Christidis P, Ioannidis O*, Loutzidou L (George Papanikolaou General Hospital of Thessaloniki, Thessaloniki); Karaitianos I. G.*, Geroukalis A. (Henry Dunant Hospital Center), Tsirlis T ); Baili E, Charalabopoulos A, Liakakos T, Schizas D*, Spartalis E, Syllaios A, Zografos C (Laiko University Hospital, Athens); Christou C, Papadopoulos V, Tooulias A, Tsoulfas G* (Papageorgiou General Hospital, Thessaloniki); Athanasakis E, Chrysos E, Tsiaoussis I, Xenaki S*, Xynos E* (University Hospital of Heraklion Crete and Interclinic Hospital of Crete, Heraklion Crete).

Hong Kong: Futaba K*, Ho MF, Hon S, Mak TWC, Ng S (Prince of Wales Hospital, Sha Tin); Foo CC* (Queen Mary Hospital, Pok Fu Lam).

Hungary: Banky B*, Suszták N (Szent Borbála Kórház, Tatabánya).

India: Misra S*, Pareek P, Vishnoi JR* (All India Institute of Medical Sciences (Aiims), Jodhpur, Jodhpur); Jain A, Mishra S, Mishra T, Mitra JK, Muduly D* (All India Institute of Medical Sciences, Bhubhaneshwar); Agrawal A, Garg PK, Kottayasamy Seenivasagam R*, Majumdar KS, Mishra N, Singh MP (All India Institute of Medical Sciences, Rishikesh); Chyau Patnaik S, Rao S*, Reddy P, S RRR, Saksena AR, Y J (Basavatarakam Indo American Cancer Hospital and Research Institute, Hyderabad); Ayloor Seshadri R* (Cancer Institute (Wia) Regional Cancer Centre, Chennai); Haque PD*, Jeyaraj P, Kannummal Veetil S, Mahajan A (Christian Medical College and Hospital, Ludhiana); Devarakonda S, Jesudason MR, Mittal R*, Moorthy M, Yezzaji H (Christian Medical College and Hospital, Vellore); Aggarwal M, Dhamija P, Kumar A* (Government Medical College Patiala, Patiala); Chisthi M, G D, George G, Kollengode VV*, Kuttanchettiyar KG, Yadev I* (Government Medical College Thiruvananthapuram, Thiruvananthapuram); Dharanipragada K*, Kalayarasan R*, Penumadu P* (Jawaharlal Institute of Postgraduate Medical Education and Research, Pondicherry); L B, Mathew S* (Kasturba Medical College Hospital, Manipal, Manipal); Akhtar N, Chaturvedi A, Gupta S, Kumar V, Rajan S* (King George’s Medical University, Lucknow); Agrawal N, Arora A, Chaturvedi H, Jain M, Kumar S*, Singh S (Max Superspeciality Hospital, New Delhi); Bhat GA, Chowdri N, Mehraj A*, Parray F Q, Shah ZA, Wani R (Sher‐I‐Kashmir Institute of Medical Sciences, Srinagar); Ahmed Z, Bhat MA, Laharwal A, Mahmood M, Mir I, Mohammad Z, Muzamil J, Rashid A*, Singh R (Smhs Hospital, Government Medical College, Srinagar); Ahmed A, Jain D, Pipara A (Tata Medical Center, Kolkata); Desoouza A, Pandey D, Pramesh CS*, Saklani A (Tata Memorial Hospital, Mumbai).

Indonesia: Islam AA*, Kembuan G, Pajan H (Rsud Wahidin Sudirohusodo, Makassar).

Ireland: Aremu M*, Canas‐Martinez A, Cullivan O, Murphy C, Owens P, Pickett L (Connolly Hospital Blanchardstown, Dublin); Corrigan M*, Daly A, Fleming CA*, Jordan P, Kayyal MY, Killeen S, Lynch N, O’Brien S, Syed WAS, Vernon L (Cork University Hospital, Cork); Hanly A, Heneghan H*, Kennelly R, Martin ST, Winter D* (St Vincent’s University Hospital, Dublin); Fahey BA, Larkin J*, Mccormick P, Mehigan BJ, Mohan H, Shokuhi P, Smith J (St James’s Hospital, Dublin); Bashir Y, Bass G, Connelly T, Creavin B, Earley H, Elliott JA*, Gillis AE, Kavanagh D, Neary P, O’Riordan J, Reynolds IS, Rice D, Ridgway PF, Umair M, Whelan M (Tallaght University Hospital, Dublin); Corless K, Finnegan L, Fowler A, Hogan A, Lowery A*, McKevitt K*, Nugent E, Ryan É J (University Hospital Galway, Galway); Coffey JC, Cunningham RM, Devine M, Nally D*, Peirce C (University Hospital Limerick, Limerick); Hardy NP, Neary PM, O’Malley S*, Ryan M (University Hospital Waterford/University College Cork, Waterford).

Israel: Gaziants V, Gold‐ Deutch R, Lavy R, Zmora O* (Shamir Medical Center, Be’er Ya’akov).

Italy: Macina S* (Asst Mantua, Mantova); Mariani NM*, Opocher E, Pisani Ceretti A (Asst Santi Paolo E Carlo, Milan); Bianco F* (Asst‐Papa Giovanni Xxiii‐ Bergamo, Bergamo); Marino M*, Marino MV*, Mirabella A, Vaccarella G (Azienda Ospedaliera Ospedali Riuniti Villa Sofia‐Cervello, Palermo, Palermo); Sena G* (Azienda Ospedaliera Pugliese‐Ciaccio Di Catanzaro, Catanzaro); Agostini C, Alemanno G, Bartolini I, Bergamini C, Bruscino A, De Vincenti R, Di Bella A, Fortuna L, Maltinti G, Muiesan P*, Prosperi P*, Ringressi MN, Risaliti M, Taddei A*, Tucci R (Azienda Ospedaliera Universitaria Careggi, Firenze); Campagnaro T*, Guglielmi A, Pedrazzani C, Rattizzato S, Ruzzenente A, Turri G (Azienda Ospedaliera Universitaria Integrata Di Verona, Verona); Altomare DF, Papagni V, Picciariello A* (Azienda Ospedaliero Universitaria Consorziale Policlinico Di Bari, Bari); Bellora P, D’Aloisio G, Ferrari M, Francone E, Gentilli S*, Nikaj H (Azienda Ospedaliero Universitaria Maggiore Della Carità, Novara); Bianchini M, Chiarugi M, Coccolini F, Cremonini C, Di Franco G, Furbetta N, Gianardi D, Guadagni S, Morelli L*, Musetti S, Palmeri M, Tartaglia D* (Azienda Ospedaliero Universitaria Pisana, Pisa); Anania G*, Carcoforo P*, Chiozza M, De Troia A, Koleva Radica M, Portinari M, Sibilla MG, Urbani A (Azienda Ospedaliero Universitaria Sant’anna, Ferrara); Fabbri N, Feo CV*, Gennari S, Parini S, Righini E (Azienda Unità Sanitaria Locale di Ferrara ‐Università di Ferrara, Ferrara); Annessi V, Castro Ruiz C, Giunta A, Montella MT, Zizzo M* (Azienda Unità Sanitaria Locale—IRCCSDi Reggio Emilia, Reggio Emilia); Grossi U, Novello S, Romano M, Rossi S, Zanus G* (Ca’ Foncello Treviso—DiSCOG—Università Di Padova, Treviso); Esposito G, Frongia F, Pisanu A, Podda M* (Cagliari University Hospital, Cagliari); Belluco C, Lauretta A*, Montori G, Moras L, Olivieri M (Centro Di Riferimento Oncologico Di Aviano (Cro) Irccs, Aviano); Feo CF, Perra T*, Porcu A*, Scanu AM (Cliniche San Pietro, A.o.u. Sassari, Sassari); Aversano A, Carbone F, Delrio P*, Di Lauro K, Fares Bucci A, Rega D*, Spiezio G (Colorectal Surgical Oncology Unit—Istituto Nazionale Tumori Fondazione, Pascale—I.R.C.C.S., Naples); Pirozzolo G*, Recordare A, Vignotto C (Dell’angelo Hospital, Venezia); Calabrò M*, Farnesi F, Lunghi EG, Muratore A*, Pipitone Federico NS (Edoardo Agnelli, Pinerolo); De Palma G, Luglio G*, Pagano G, Tropeano FP (Federico Ii University Hospital, Naples); Baldari L*, Boni L*, Cassinotti E* (Department of Surgery Fondazione IRCCS Ca’ Granda—Ospedale Maggiore Policlinico, Milan); Cosimelli M, Fiore M*, Guaglio M*, Sorrentino L (Fondazione IRCCS Istituto Nazionale dei Tumori, Milano); Agnes A, Alfieri S, Belia F, Biondi A, Cozza V, D’Ugo D, De Simone V, Litta F, Lorenzon L, Marra AA, Marzi F, Parello A, Persiani R, Ratto C, Rosa F, Scrima O, Sganga G (Fondazione Policlinico Universitario Agostino Gemelli IRCCS, Rome); Belli A*, Izzo F, Patrone R (Hpb Surgical Oncology Unit—Department of Abdominal Oncology, Istituto Nazionale Tumori‐IRCCS ‘Fondazione G. Pascale’, 80131 Naples, Italy.); Foppa C, Carvello MM, De Lucia F, Spinelli A* (Department of Biomedical Sciences, Humanitas University, Via Rita Levi Montalcini 4, 20090 Pieve Emanuele, Milan, Italy; IRCCS Humanitas Research Hospital, via Manzoni 56, 20089 Rozzano, Milan, Italy); Aprile A, Batistotti P, Massobrio A, Pertile D, Scabini S*, Soriero D (IRCCS Ospedale Policlinico San Martino, Genoa); De Manzoni Garberini A* (Ospedale Civile Spirito Santo, Pescara); Mazzotti F*, Pasini F, Ugolini G (Ospedale Degli Infermi Di Faenza, Faenza); Barone R, Birolo SL*, Caccetta M, Deirino A, Garino M, Grasso M, Marafante C, Masciandaro A, Moggia E, Mungo S, Murgese A, Raggio E (Ospedale Degli Infermi Di Rivoli, Rivoli); Federico P, Maida P, Marra E, Marte G, Petrillo A, Tammaro P, Tufo A* (Ospedale Del Mare, Naples); Berselli M*, Borroni G*, Cocozza E, Conti L, Desio M, Rizzi A (Ospedale Di Circolo, University of Insubria, University Hospital of Varese, Asst Sette Laghi, Regione Lombardia, Varese Lombardy); Baldi C*, Corbellini C, Sampietro GM (Ospedale Di Rho—Asst Rhodense, Rho); Bordoni P, Clarizia G, Fleres F*, Franzini M, Grechi A, Longhini A, Spolini A (Ospedale Di Sondrio (Asst Valtellina E Alto Lario), Sondrio); Baldini E*, Capelli P, Conti L, Isolani SM, Ribolla M (Ospedale Guglielmo Da Saliceto—Piacenza, Piacenza); Bondurri A, Colombo F*, Ferrario L, Guerci C, Maffioli A (Ospedale Luigi Sacco Milano, Milan); Armao T, Ballabio M*, Bisagni P*, Longhi M, Madonini M, (Ospedale Maggiore Di Lodi, Lodi); Impellizzeri H, Inama M*, Moretto G (Ospedale Pederzoli, Verona); Mochet S*, Usai A (Ospedale Regionale Umberto Parini, Aosta); Bianco F*, Incollingo P (Ospedale S. Leonardo—Asl Napoli 3 Sud, Castellammare Di Stabia, Naples); Giacometti M*, Zonta S (Ospedale San Biagio, Asl Vco, Domodossola); Marino Cosentino L*, Sagnotta A* (Ospedale San Filippo Neri, Rome); Nespoli LC, Tamini N* (Ospedale San Gerardo, Monza); Anastasi A, Bartalucci B, Bellacci A, Canonico G*, Capezzuoli L, Di Martino C, Ipponi P, Linari C, Montelatici M, Nelli T, Spagni G, Tirloni L, Vitali A (Ospedale San Giovanni Di Dio, Firenze); Abate E, Casati M*, Laface L, Schiavo M (Ospedale Vittorio Emanuele III—Carate Brianza, Carate Brianza (MB)); Arminio A, Cotoia A, Lizzi V*, Vovola F (Ospedali Riuniti Azienda Ospedaliera Universitaria Foggia, Foggia); Vergari R* (Ospedali Riuniti Di Ancona, Ancona); D’Ugo S*, Depalma N, Spampinato MG (Ospedale ‘Vito Fazzi’, Lecce); Annicchiarico A, Catena F*, Giuffrida M, Perrone G (Parma University Hospital, Parma); Baronio G, Carissimi F, Montuori M, Pinotti E* (Policlinico San Pietro, Ponte San Pietro); Brachini G, Chiappini A, Cicerchia PM, Cirillo B, De Toma G, Fiori E, Fonsi GB, Iannone I, La Torre F, Lapolla P*, Meneghini S, Mingoli A, Sapienza P, Zambon M (Policlinico Umberto I Sapienza University of Rome, Rome); Capolupo GT*, Carannante F, Mazzotta E (Policlinico Universitario Campus Bio Medico of Rome, Rome); Gattolin A, Migliore M, Rimonda R, Sasia D*, Travaglio E (Regina Montis Regalis Hospital, Mondovì); Chessa A*, Fiorini A, Norcini C (San Giovanni Di Dio, Orbetello); Colletti G, Confalonieri M, Costanzi A*, Frattaruolo C, Mari G, Monteleone M, Locatelli A, Riva C, Balconi A (San Leopoldo Mandic Hospital, Merate, ASST Lecco); De Nardi P*, Parise P, Vignali A (San Raffaele Scientific Institute, Milan,); Belvedere A, Bernante P, Droghetti M, Jovine E, Neri J, Parlanti D, Pezzuto AP, Poggioli G, Rottoli M*, Russo IS, Tanzanu M, Violante T (, Alma Mater Studiorum University of Bologna, Italy, Bologna; Borghi F, Cianflocca D, Di Maria Grimaldi S, Donati D, Gelarda E, Giraudo G, Giuffrida MC, Marano A*, Palagi S, Pellegrino L, Peluso C, Testa V* (Santa Croce E Carle Hospital, Cuneo, Cuneo); Agresta F*, Prando D*, Zese M* (Santa Maria Degli Angeli Hospital Ulss5—Adria, Adria); Armatura G*, Frena A, Bertelli G, Marinello P, Notte F, Scotton G* (, Bolzano Central Hospital); Cervellera M, Gori A, Sartarelli L, Tonini V* (S. Orsola‐Malpighi Hospital, Bologna); Gallo G*, Sammarco G, Vescio G (University ‘Magna Graecia’ of Catanzaro, Catanzaro); Di Marzo F* (Valtiberina, Sansepolcro). Rega D*, Delrio P* (Colorectal Surgical Oncology, Department of Abdominal Oncology, Istituto Nazionale Tumori‐IRCCS ‘Fondazione G. Pascale’, 80131

Japan: Kanemitsu Y*, Moritani K (National Cancer Center Hospital, Tokyo).

Jordan: Al Abdallah M*, Ayasra F, Ayasra Y, Qasem A (Al‐Basheer Hospital, Amman); Fahmawee T, Hmedat A, Obeidat K* (King Abdullah University Hospital, Ar Ramtha); Abou Chaar MK, Al‐Masri M*, Al‐Najjar H, Alawneh F (King Hussein Cancer Center, Amman).

Libya: Aldokali N, Senossi O, Subhi MT (Alkhadra Hospital, Tripoli); Algallai M, Alwarfly S, AlZAEDE S, Gahwagi M*, Moftah M (Benghazi Medical Center, Benghazi); Burgan D*, Kamoka E, Kilani AI (National Cancer Institute, Sabratha—Libya, Sabratha); Abdelkabir M*, Altomi I (Sabha Medical Center, Tripoli); Abdulwahed E*, Alshareea E, Aribi N, Aribi S, Biala M, Ghamgh R (Tripoli Central Hospital, Tripoli); Alsoufi A, Ellojli I*, Kredan A, Msherghi A* (Tripoli University Hospital, Tripoli); Alshareef K* (Yashfeen Clinic, Tajora‐Tripoli); Ghadah Z Alkadiki, Faraj S Almaadany, (AlJalaa Hospital Benghazi).

Lithuania: Bradulskis S, Dainius E, Kubiliute E, Kutkevičius J, Parseliunas A, Subocius A, Venskutonis D* (Lithuanian University of Health Sciences. LUHS Kaunas Hospital, Kaunas).

Madagascar: Rasoaherinomenjanahary F*, Razafindrahita JB, Samison LH (Joseph Ravoahangy Andrianavalona Hospital, Antananarivo).

Malaysia: Ngo CW*, Ramasamy S (Hospital Enche’ Besar Hajjah Khalsom, Kluang, Johor); Hamdan KH, MdRazali.I, Tan JA, Thanapal MR* (Hospital Kuala Lumpur, Kuala Lumpur); Choong E, Lim RZM (Hospital Sultanah Aminah, Batu Pahat, Johor); Nik Amin Sahid, Hayati F*, Jayasilan J, Sriram RK*, Subramaniam S (Queen Elizabeth Hospital and Universiti Malaysia Sabah, Kota Kinabalu, Sabah, Malaysia); Ibrahim AF* (Sarawak General Hospital, Kuching, Sarawak); Che jusoh A, Hussain AH, Mohamed Sidek AS, Mohd Yunus MF, Soh JY, Wong MP, Zakaria AD*, Zakaria Z (School of Medical Sciences and Hospital, Universiti Sains Malaysia, Kelantan); Mohd Azman ZA (University Kebangsaan Malaysia Medical Centre, Kuala Lumpur); Fathi NQ, Roslani AC*, Xavier R (University Malaya Medical Centre, Kuala Lumpur and Universiti Putra Malaysia).

Mexico: Alvarez MR, Cordera F*, Hernandez R (Abc Medical Center, Mexico City); Soulé Martínez CE* (Hospital Central Norte Pemex, Mexico City); Aboharp Hasan Z, Otoniel LR, Sosa Duran EE* (Hospital Juárez De México, Ciudad De México); Melchor‐Ruan J*, Romero Bañuelos E, Vilar‐Compte D (Instituto Nacional De Cancerologia, Mexico City); Buerba GA, Mercado MÁ*, Posadas‐Trujillo OE, Salgado‐Nesme N, Sarre C (Instituto Nacional De Ciencias Médicas Y Nutrición ‘Salvador Zubirán’, Mexico City).

Morocco: Amrani L, Benkabbou A, El Ahmadi B, El Bouazizi Y, Belkhadir ZH, Ghannam A, Majbar AM, Mohsine R, Souadka A* (Université mohammed V, Institut National D’oncologie, Rabat).

Netherlands: Hompes R*, Meima‐van Praag EM, Pronk AJM, Sharabiany S (Amsterdam UMC, University of Amsterdam, Amsterdam); Grotenhuis BA*, Hartveld L (Antoni Van Leeuwenhoek Ziekenhuis, Amsterdam); Ebben L, Kuiper S*, Melenhorst J, Poeze M (Mumc+, Maastricht); Posma‐Bouman L* (Slingeland Ziekenhuis, Doetinchem); Derksen T, Franken J, Oosterling S* (Spaarne Gasthuis, Haarlem); Konsten J*, Van Heinsbergen M (Viecuri Medisch Centrum, Venlo).

Nigeria: Adeyeye A, Enoch E (Afe Babalola University Multi‐System Hospital, Ido Ekiti); Nwabuoku SE, Sholadoye TT*, Tolani MA* (Ahmadu Bello University Teaching Hospital, Zaria); Olaogun J* (Ekiti State University Teaching Hospital, Ado‐Ekiti); Abiyere H, Babalola O, Okunlola A* (Federal Teaching Hospital, Ido Ekiti, Ido Ekiti); Ali SANI S, Chinda J, Garba S, Mshelbwala P, Olori S*, Olute A, Osagie O, Pius Ogolekwu I, Umar A (University of Abuja Teaching Hospital, Gwagwalada); Abdur‐Rahman LO*, Adeyeye A, Bello JO, Olasehinde O, Popoola AA (University of Ilorin Teaching Hospital, Ilorin).

Pakistan: Abassy J*, Ahmed K, Alvi A, Khan S*, Pirzada A, Saleem A, Siddiqui MT, Turk K (Aga Khan University, Karachi); Jamal A, Kerawala AA* (Cancer Foundation Hospital, Karachi); Memon AS*, Nafees Ahmed R, Rai L* (Dr Ruth K.m. Pfau Civil Hospital, Karachi); Ali AA* (King Edward Medical University, Mayo Hospital, Lahore, Lahore); Afzal MF, Khokhar MI* (Lahore General Hospital, Pgmi, Amc, Lahore); Ayub B, Ramesh P, Sayyed R* (Patel Hospital, Karachi); Ayyaz M*, Butt U*, Kashif M, Qureshi AU*, Farooka MW* (Services Hospital Lahore, Lahore); Ayubi A, Waqar SH* (The Pakistan Institute of Medical Sciences, Islamabad).

Palestine: Al‐Slaibi I, I. A. Alzeerelhouseini H, Jobran F* (Al‐Ahli Hospital, Hebron, West Bank); Abukhalaf SA (Palestine Medical Complex, Ramallah, West Bank).

Panama: Cukier M* (Pacifica Salud Hospital, Panama).

Philippines: Jocson R, Teh C*, Uy Magadia E (National Kidney and Transplant Institute, Quezon City).

Poland: Major P (Jagiellonian University Medical College, Krakow); Bąk M, Dubieńska K, Ławnicka A, Murawa D* (Karol Marcinkowski University Hospital, Zielona Gora); Janik M, Kowalewski P, Kwiatkowski A, Roszkowski R, Sroczyński P, Walędziak M* (Military Institute of Medicine, Warsaw).

Portugal: Azevedo C, Machado D, Mendes F* (Centro Hospitalar Cova Da Beira, Covilhã); De Sousa X* (Centro Hospitalar De Setúbal, Setúbal); Fernandes U, Ferreira C*, Guidi G, Marçal A, Marques R, Martins D, Vaz‐Pereira R, Vieira B (Centro Hospitalar De Trás‐Os‐Montes E Alto Douro, E.p.e., Vila Real); Almeida JI, Almeida‐Reis R*, Correia de Sá T, Costa MJMA, Fernandes V, Ferraz I, Lima da Cruz L, Lima da Silva C, Lopes L, Machado N, Marialva J, Nunes Coelho M, Pedro J, Pereira C, Ribeiro A, Ribeiro CG, Santos R, Saraiva P, Silva R, Tavares F, Teixeira M (Centro Hospitalar do Tâmega e Sousa, Penafiel); Almeida AC, Amaral MJ, Andrade R, Camacho C, Costa M, Lázaro A*, Nogueira O, Oliveira A, Ruivo A, Silva M, Simões JFF (Centro Hospitalar E Universitário De Coimbra, Coimbra); Devezas V, Jácome F, Nogueiro J, Pereira A, Santos‐Sousa H*, Vaz S (Centro Hospitalar E Universitário De São João, Porto); Pinto J, Tojal A* (Centro Hospitalar Tondela‐Viseu, Viseu); Cardoso P*, Martins R*, Martins dos Santos G, Henriques P, Morais H, Sousa S, Cardoso N, Teixeira J, Pereira R, Revez T, Ribeiro R, Manso I, Domingues J (Centro Hospitalar Universitário Do Algarve—Unidade De Faro, Faro); Amorim E, Baptista VH, Cunha MF*, Sampaio da Nóvoa Gomes Miguel II (Centro Hospitalar Universitário Do Algarve—Unidade De Portimão, Portimão); Bandovas JP, Borges N*, Chumbinho B, Figueiredo de Barros I, Frade S, Gomes J, Kam da Silva Andrade A, Pereira Rodrigues A, Pina S, Silva N*, Silveira Nunes I, Sousa R (Centro Hospitalar Universitário Lisboa Central, Lisbon); Azevedo P, Costeira B, Cunha C, Garrido R*, Miranda P, Peralta Ferreira M, Sousa Fernandes M (Hospital Beatriz Angelo, Loures); Azevedo J* (Hospital Da Horta, E.p.e., Horta); Galvão D, Vieira A* (Hospital De Santo Espirito Da Ilha Terceira, Angra Do Heroismo); Patrício B, Santos PMDD*, Vieira Paiva Lopes AC (Hospital De Torres Vedras—Centro Hospitalar Do Oeste, Torres Vedras); Cunha R, Faustino A, Freitas A, Mendes JR*, Parreira R (Hospital Do Divino Espírito Santo, Ponta Delgada); Abreu da Silva A*, Claro M, Costa Santos D, Deus AC, Grilo JV (Hospital Do Litoral Alentejano, Santiago Do Cacém); Castro Borges F*, Corte Real J, Henriques S, Lima MJ, Matos Costa P (Hospital Garcia De Orta, Almada); Alagoa Joao A, Azevedo P, Camarneiro R, Capunge I, Fragoso M, Frazão J, Martins A, Pedro V, Pera R, Ramalho de Almeida F, Sampaio Soares A*, Vale R, Vasconcelos M (Hospital Prof. Doutor Fernando Fonseca, E.p.e., Amadora); Brito da Silva F, Caiado A*, Fonseca F (Instituto Português De Oncologia De Lisboa Francisco Gentil, Lisboa); Ângelo M, Baião JM, Martins Jordão D*, Vieira Caroço T (Ipo Coimbra, Coimbra); Baía C, Canotilho R, Correia AM, Ferreira Pinto AP, Peyroteo M, Videira JF* (Ipo Porto, Porto).

Réunion: Kassir R*, Sauvat F (Chu Reunion, Saint Denis).

Romania: Bonci E*, Gata V*, Titu S* (‘Prof. Dr. Ion Chiricuta’ Institute of Oncology, Cluj‐Napoca); Bezede C, Chitul A, Ciofic E, Cristian D, Grama F* (Coltea Clinical Hospital, Bucharest); Ciubotaru C, Negoi I*, Negoita VM, Stoica B (Emergency Clinical Hospital Bucharest, Bucharest); Ginghina O*, Iordache N, Iosifescu RV, Mardare M, Mirica RM, Spanu A, Văcărașu AB, Zamfir‐Chiru‐Anton M (Saint John Emergency Hospital, Bucharest).

Russia: Tsarkov P*, Tulina I (Clinic of Coloproctology and Minimally Invasive Surgery, Sechenov Medical State University, Moscow); Abelevich A, Bazaev A, Kokobelyan AK, Yanishev A* (Cprivolzhsky Research Medical University, Nizhny Novgorod Regional Clinical Hospital, Sechenov Medical State University, Nizhny Novgorod); Litvin A*, Litvina Y, Provozina A (Immanuel Kant Baltic Federal University, Regional Clinical Hospital, Kaliningrad); Agapov M*, Garmanova T, Kazachenko E, Markaryan D, Galliamov E, Kakotkin V, Kubyshkin V, Semina E, Kamalov A (Moscow Research and Educational Center, Lomonosov Moscow State University, Moscow); Novikova A, Zakharenko A* (Pavlov First State Medical University of St Petersburg, Saint Petersburg).

Saudi Arabia: Alshahrani M*, Alsharif F, Eskander M (Aseer Central Hospital, Abha); Majrashi S*, Mashat A (East Jeddah General Hospital, Jeddah); Alharthi M, Aljiffry M, Basendowah M, Malibary N*, Nassif M, Saleem A, Samkari A, Trabulsi N* (King Abdulaziz University Hospital, Jeddah); A Azab M* (King Abdullah Medical City Makkah, Makkah); Alqahtani D, Jaloun H*, Mudawi I (King Fahad Armed Forces Hospital, Jeddah); Al Awwad S*, Alghamdi M*, Alnumani T* (King Fahad General Hospital, Jeddah); Awad S*, I Sharara M (King Faisal Medical Complex, Taif City); Al Habes H, Alqannas M*, Alyami M*, Alzamanan M, Cortés‐Guiral D*, Elawad A (King Khalid Hospital, Najran); Adi H, Al ahmad F, Al Ayed A, Alishi Y (King Salman Armed Forces Hospital, Tabouk); AlAamer O, Alselaim N* (King Saud Bin Abdulaziz University For Health Sciences, King Abdullah International Medical Research Center, Ministry of National Guard, Health Affairs, General Surgery Department., Riyadh); Alfaifi J, D’Souza J (King Saud Medical City, Riyadh); Al‐Khayal K, Alhassan N, Alobeed O, Alshammari S, Bin Nasser A*, Bin Traiki T, Nouh T*, Zubaidi A (King Saud University, Riyadh); Abdulfattah F, Al‐Kharashi E*, Alanazi F, Albaqami F, Alghuliga A, Alsowaina K, Arab N*, Badahdah F (Prince Sultan Military Medical City, Riyadh); Alobaysi S, Alshahrani A, Alzahrani A* (Security Forces Hospital, Riyadh).

Serbia: Aleksić L, Antic A, Barisic G*, Ceranic M, Grubač Ž, Jelenkovic J, Kecmanović D, Kmezić S, Knezevic D*, Krivokapic Z*, Latinčić S, Markovic V*, Matić S*, Miladinov M, Pavlov M*, Pejovic I, Tadic B, Vasljević J, Velickovic D (Clinic For Digestive Surgery, Clinical Centre of Serbia, Belgrade); Doklestic K, Gregoric P*, Ivancevic N, Loncar Z, Micic D (Clinic For Emergency Surgery, Emergency Centre, Clinical Centre of Serbia, Belgrade); Buta M, Cvetkovic A, Gacic S, Goran M, Jeftic N, Markovic I*, Milanović M, Nikolic S, Pejnovic L, Savković N, Stevic D, Vucic N, Zegarac M (Institute For Oncology and Radiology of Serbia, Belgrade); Karamarkovic A, Kenic M, Milic Lj, Kovacevic B, Krdzic I* (Zvezdara University Medical Center, Belgrade).

Singapore: Lieske B* (National University Hospital, Singapore).

South Africa: Almgla N*, Boutall A, Herman A, Kloppers C*, Nel D, Rayamajhi S (Groote Schuur Hospital, Cape Town).

Spain: Paniagua García Señorans M*, Vigorita V (Álvaro Cunqueiro Hospital, Vigo); Acrich E, Baena Sanfeliu E, Barrios O, Golda T*, Santanach C, Serrano‐Navidad M, Sorribas Grifell M, Vives RV (Bellvitge University Hospital, Hospitalet De Llobregat); Escolà D, Jiménez A* (Comarcal Alt Penedés, Barcelona); Alcázar JA, Angoso‐Clavijo M, Blanco‐Antona F, Carabias‐Orgaz A, Díaz Maag R, Garcia J, García‐Plaza AGP, Gonzalez‐Muñoz JI, Muñoz‐Bellvis L, Sánchez Tocino JM, Sanchez‐Casado AB, Trebol J* (Complejo Asistencial Universitario De Salamanca, Salamanca); Hernandez Gutierrez J, Tébar Zamora A* (Complejo Hospitalario De Toledo, Toledo); Palma P, Cayetano Paniagua L, Gomez Fernandez L* (Consorci Sanitari De Terrassa, Barcelona); Collera Ormazabal P, Diaz del Gobbo R, Farre Font R, Flores Clotet R, Gómez Díaz CJ*, Guàrdia N, Guariglia CA, Osorio Ramos A, Sanchez Jimenez R, Sanchon L, Soto Montesinos C (Fundació Althaia—Xarxa Assistencial Universitària de Manresa, Manresa); Alonso‐Lamberti L, García‐Quijada J, Jimenez Miramón J, Jimenez V*, Jover JM, Leon R, Rodriguez JL, Salazar A, Valle Rubio A (Getafe University Hospital, Getafe); Aguado López H* (Hellín Hospital, Albacete); Bravo Infante R, De Lacy FB, Lacy AM*, Otero A, Turrado‐Rodriguez V*, Valverde S (Hospital Clinic Barcelona, Barcelona); Anula R, Avellana R, Camarero Rodríguez E, Catalán Garza V, Dziakova J, García Alonso M, Lasses Martínez B, López Antoñanzas L, Muguerza JM*, Ochagavía S, Peña Soria MJ, Rivera‐Alonso D, Saez Carlin P, Sánchez del Pueblo C, Sanz Ortega G, Sanz‐López R, Torres A (Hospital Clínico De Madrid, Madrid); Martín‐Arévalo J, Moro‐Valdezate D*, Pla‐Marti V (Hospital Clínico Universitario De Valencia, Valencia); Beltrán de Heredia J, De Andrés‐Asenjo B*, Gómez Sanz T, Jezieniecki C, Nuñez Del Barrio H, Ortiz de Solórzano Aurusa FJ, Romero de Diego A, Ruiz Soriano M, Trujillo Díaz J, Vázquez Fernández A (Hospital Clínico Universitario De Valladolid, Valladolid); Lora‐Cumplido P, Sosa‐Rodríguez MV* (Hospital De Cabueñes, Gijón); Galvan Perez A, Gonzalez‐Gonzalez E, Minaya Bravo AM*, San Miguel C (Hospital Del Henares, Madrid); Alonso de la Fuente N, Jimenez Toscano M* (Hospital Del Mar, Barcelona); Grau‐Talens EJ, Martin‐Perez B* (Hospital Don Benito‐Villanueva, Don Benito (Badajoz)); Benavides Buleje JA, Carrasco Prats M*, Giménez Francés C*, Muñoz Camarena JM, Parra Baños PA, Peña Ros E, Ramirez Faraco M, Ruiz‐Marín M*, Valero Soriano M (Hospital General Reina Sofía, Murcia); Allue M, Colsa P, García Domínguez M, Gimenez Maurel T, Martín Anoro LF, Ponchietti L, Rodriguez Artigas JM, Roldón Golet M*, Utrilla Fornals A (Hospital General San Jorge, Huesca); Estaire Gómez M*, Fernández Camuñas Á, Garcia Santos EP, Jimenez Higuera E, Martínez‐Pinedo C, Muñoz‐Atienza V, Padilla‐Valverde D*, Picón Rodríguez R, Sánchez‐García S, Sanchez‐Pelaez D (Hospital General Universitario De Ciudad Real, Ciudad Real); Curtis Martínez C, Sánchez‐Guillén L* (Hospital General Universitario De Elche, Elche); Colombari RC, Del valle E, Fernández M, Lozano Lominchar P*, Martín L, Rey Valcarcel C, Zorrilla Ortúzar J (Hospital General Universitario Gregorio Marañón, Madrid); Alcaide Matas F, García Pérez JM, Troncoso Pereira P* (Hospital Mateu Orfila, Mahon); Blas Laina JL, Cros B, Escartin J*, Garcia Egea J, Nogués A, Talal El‐Abur I, Yánez C (Hospital Royo Villanova, Zaragoza); Mora‐Guzmán I* (Hospital Santa Bárbara, Puertollano); Achalandabaso Boira M*, Sales Mallafré R (Hospital Universitari De Tarragona Joan XXIII, Tarragona); Marín H, Prieto Calvo M, Villalabeitia Ateca I* (Hospital Universitario Cruces, Barakaldo); De Andres Olabarria U, Durán Ballesteros M, Fernández Pablos FJ, Ibáñez‐Aguirre FJ, Sanz Larrainzar A, Ugarte‐Sierra B* (Hospital Universitario De Galdakao, Galdakao‐Usansolo); Acosta Mérida MA*, Ortiz López D, Yepes Cano AF (Hospital Universitario De Gran Canaria Doctor Negrín, Las Palmas De Gran Canaria); Correa Bonito A, Delgado Búrdalo L, Di Martino M*, García Septiem J*, Maqueda González R, Martin‐Perez E (Hospital Universitario De La Princesa, Madrid); Calvo Espino P*, Guillamot Ruano P (Hospital Universitario De Móstoles, Móstoles); Colao García L, Díaz Pérez D*, Esteban Agustí E, Galindo Jara P, Gutierrez Samaniego M*, Hernandez Bartolome MA*, Serrano González J (Hospital Universitario De Torrejón De Ardoz, Madrid); Alonso Poza A, Diéguez B, García‐Conde M, Hernández‐García M, Losada M* (Hospital Universitario Del Sureste, Madrid); Alvarez E, Chavarrias N, Gegúndez Simón A, Gortázar de las Casas S, Guevara‐Martínez J, Prieto Nieto MI, Ramos‐Martín P, Rubio‐Perez I*, Saavedra J, Urbieta A (Hospital Universitario La Paz, Madrid); Aparicio‐López D, Cantalejo diaz M, De Miguel Ardevines MDC, Dobón Rascón MÁ, Duque‐Mallén V*, Gascon Ferrer I, González‐Nicolás Trébol MT, Gracia‐Roche C, Herrero Lopez M, Kälviäinen H, Martinez German A, Matute M, Sánchez Fuentes N, Santero‐Ramirez MS, Saudí S (Hospital Universitario Miguel Servet, Zaragoza); Blazquez Martin A, Diez Alonso M*, Hernandez P, Mendoza‐Moreno F, Ovejero Merino E, Vera Mansilla C, Matías‐García B, Quiroga‐Valcárcel A (Hospital Universitario Principe De Asturias, Madrid); Barranquero AG, Cerro Zaballos C, Núñez J, Ocaña J, Ramos D* (Hospital Universitario Ramón Y Cajal, Madrid); Acebes García F, Bailon‐Cuadrado M, Bueno Cañones AD, Choolani Bhojwani E, Marcos‐Santos P, Miguel T, Pacheco Sánchez D, Pérez‐Saborido B, Sanchez Gonzalez J, Tejero‐Pintor FJ* (Hospital Universitario Río Hortega, Valladolid); Alconchel F*, Conesa A, Gil Martínez J*, Gutiérrez Fernández AI, Lopez Abad A, Nicolás‐López T*, Ramirez Romero P, Roca Calvo MJ, Rodrigues K*, Ruiz Manzanera JJ, Soriano AI (Hospital Universitario Virgen De La Arrixaca, Murcia); Cano A, Capitan‐Morales L, Cintas Catena J, Gomez‐Rosado J*, Oliva Mompean F, Pérez Sánchez MA, Río Lafuente FD, Torres Arcos C, Valdes‐Hernandez J (Hospital Universitario Virgen Macarena, Seville); Cholewa H, Frasson M, Martínez Chicote C, Sancho‐Muriel J* (Hospital Universitario Y Politécnico La Fe, Valencia); Estraviz B, Fernández Gómez Cruzado L, González de Miguel M, Landaluce‐olavarria A* (Hospital Urduliz, Bizkaia); Abad‐Gurumeta A, Abad‐Motos A, Martínez‐Hurtado E, Ripollés‐Melchor J*, Ruiz‐ Escobar A (Infanta Leonor University Hospital, Madrid); Cuadrado‐García A*, Garcia‐Sancho Tellez L*, Heras Aznar J*, Maté Mate P, Ortega Vázquez I*, Picardo AL, Rojo López JA, Sanchez Cabezudo Noguera F*, Serralta de Colsa D* (Infanta Sofía University Hospital, San Sebastian De Los Reyes); Cagigas Fernandez C, Caiña Ruiz R, Gomez Ruiz M, Martínez‐Pérez P, Santarrufina Martinez S*, Valbuena Jabares V (Marqués De Valdecilla University Hospital, Santander); Cagigal Ortega EP, Cervera I, Díaz Peña P, Gonzalez J, Marqueta De Salas M, Perez Gonzalez M*, Ramos Bonilla A, Rodríguez Gómez L (Severo Ochoa University Hospital, Leganés); Alfonso Garcia M, Craus‐Miguel A, Fernández Vega L, Ferrer‐Inaebnit E, Gil Catalán A, González Argente FX, Jeri S, Oseira A, Pujol Cano N, Segura‐Sampedro JJ*, Soldevila Verdeguer C, Villalonga B (Son Espases University Hospital, Palma De Mallorca); Blanco‐Colino R, Espin‐Basany E*, Pellino G (Vall D’Hebron University Hospital, Barcelona).

Sri Lanka: Srishankar S, Thalgaspitiya SPB (Teaching Hospital, Anuradhapura)Arulanantham A, Bandara GBKD, Jayarajah U*, Ravindrakumar S, Rodrigo VSD (District General Hospital Chilaw, Chilaw).

Sudan: Ali karar AA (Al‐Rajhi, Omdurman); Elhafiz MHY* (Best Care Hospital, Khartoum); Adam Essa Adam ME*, Ahmed A (Omdurman Teaching Hospital, Khartoum); Saleh M (University of Gezira Hospital, Wad Madani).

Sweden: Arkani S*, Freedman J* (Danderyds Hospital, Stockholm); Angenete E*, Park J (Sahlgrenska University Hospital, Gothenburg); Älgå A*, Heinius G, Nordberg M, Pieniowski E (South General Hospital, Stockholm); Löfgren N, Rutegård M* (Umea University Hospital, Umea).

Switzerland: Arigoni M, Bernasconi M, Christoforidis D*, Di Giuseppe M, La Regina D, Mongelli F (Ente Ospedaliero Cantonale, Ticino (Lugano, Bellinzona, Locarno, Mendrisio)); Chevallay M, Dwidar O, Gialamas E, Sauvain M* (Hopital De Pourtales, Neuchatel); Adamina M*, Crugnale AS, Guglielmetti L, Peros G (Kantonsspital Winterthur, Winterthur); Gass M*, Metzger J, Scheiwiller A (Luzerner Kantonsspital, Luzern); Turina M* (Universitätsspital, Zürich).

Syrian Arab Republic: Al Asadi T, Alkhateb S, Altom R, Bakkar B, Maa Albared S*, Melhem S (Damascus Hospital, Damascus); Hammed A*, Hammed S (Tishreen University Hospital, Latakia).

Tunisia: Kacem MJ, Maghrebi H, Sebai A* (La Rabta Hospital, Tunis).

Turkey: Aghayeva A*, Hamzaoglu I, Sahin I (Acibadem Altunizade Hospital, Istanbul; Department of General Surgery, Acibadem Mehmet Ali Aydinlar University School of Medicine, Istanbul); Akaydin E, Aliyeva Z, Aytac E, Baca B, Ozben V*, Ozmen BB (Acibadem Atakent Hospital, Istanbul; Department of General Surgery, Acibadem Mehmet Ali Aydinlar University School of Medicine, Istanbul); Arikan AE*, Bilgin IA*, Kara H, Karahasanoglu T, Uras C (Acibadem Maslak Hospital, Istanbul; Department of General Surgery, Acibadem Mehmet Ali Aydinlar University School of Medicine, Istanbul); Akbas A, Altinel Y*, Calikoglu F, Ercan G, Ercetin C, Hacım NA, Meriç S, Tokocin M, Vartanoglu T, Yigitbas H (Bagcilar Research and Training Hospital, Istanbul); Doğangün M, Iflazoğlu N, Yalkın Ö* (Bursa City Hospital, Bursa); Cennet O, Dincer HA, Erol T (Hacettepe University Hospital, Ankara); Alhamed A, Ergün S*, Ozcelık MF, Sanli AN, Uludağ SS*, Velidedeoglu M*, Zengin AK (Istanbul University—Cerrahpaşa Medical Faculty, Istanbul); Kara Y*, Bozkurt MA, Kocatas A (Health Sciences University Kanuni Sultan Suleyman Training and Research Hospital, Istanbul); Eyuboglu K, Guner A*, Usta MA (Karadeniz Technical University Farabi Hospital, Trabzon); Azamat İF, Balik E*, Buğra D, Kulle CB (Koç University Medical School, Istanbul); Güler SA, Güreşin A, Tatar OC*, Utkan NZ, Yildirim A, Yüksel E (Kocaeli University Teaching Hospital, Kocaeli); Abbasov A*, Yanar H (Liv Hospital Ulus, Istanbul); Akin E, Altintoprak F*, Cakmak G, Çelebi F, Demir H, Dikicier E, Firat N, Gönüllü E, Kamburoğlu MB, Küçük IF, Mantoglu B (Sakarya University Faculty of Medicine, Sakarya); Çolak E*, Kucuk GO, Uyanik MS (University of Samsun, Samsun Training and Research Hospital, Samsun); Goksoy B* (Sehit Prof.dr. İlhan Varank Training and Research Hospital, Istanbul); Bozkurt E, Capkinoglu E, Guven O, Mihmanli M, Omeroglu S, Tanal M*, Yetkin G (Seyrantepe Hamidiye Etfal Training and Research Hospital, Istanbul); Akalin M, Arican C, Avci EK, Aydin C, Demirli Atici S*, Emiroglu M, Kaya T*, Kebabçı E, Kilinc Tuncer G, Kirmizi Y, Öğücü H, Salimoğlu S, Sert İ, Tugmen C, Tuncer K, Uslu G, Yeşilyurt D (University of Health Sciences Tepecik Training and Research Hospital, İzmir); Yildiz A*, Yildiz A (Yildirim Beyazit University Yenimahalle Training and Research Hospital, Ankara); Gultekin FA* (Zonguldak Bulent Ecevit University School of Medicine Research and Training Hospital, Zonguldak).

Uganda: Lule H*, Oguttu B* (Kampala International University Teaching Hospital, Ishaka).

United Kingdom: Agilinko J, Ahmeidat A, Bekheit M*, Cheung LK, Kamera BS, Mignot G, Shaikh S*, Sharma P (Aberdeen Royal Infirmary, Aberdeen); Al‐Mohammad A, Ali S, Ashcroft J, Baker O, Coughlin P, Davies RJ*, Kyriacou H, Mitrofan C, Morris A, Raby‐Smith W, Rooney SM, Singh AA, Tan XS, Townson A, Tweedle E (Addenbrooke’s Hospital, Cambridge); Kattakayam A, Lunevicius R, Sheel A, Sud A, Sundhu M (Aintree University Hospital, Liverpool); Angelou D, Choynowski M, McAree B*, McCanny A, Neely D (Antrim Area Hospital‐ Northern Health and Social Care Trust, Antrim); Kamel F, Kumar L, Madani R*, Nisar P (Ashford and St Peter’s Hospital, Chertsey); Creanga M*, Elniel M, Law J (Blackpool Victoria Hospital, Blackpool); Mosley F* (Bradford Royal Infirmary, Bradford); Arrowsmith L*, Campbell W* (Causeway Hospital, Coleraine); Grove T, Kontovounisios C, Warren O* (Chelsea and Westminster Hospital, London); Doulias T*, Li M, Martin E, Rodwell H (Colchester Hospital University, Colchester); Clifford R, Eardley N, Krishnan E, Manu N, Martin E, Roy Mahapatra S, Serevina OL, Smith C, Vimalachandran D* (Countess of Chester Hospital, Chester); Emslie K*, Labib PL*, Minto G, Natale J, Panahi P, Rogers LJ* (Derriford Hospital, Plymouth); Abubakar A*, Akhter Rahman MM, Chan E, O’Brien H, Sasapu K* (Diana Princess of Wales Hospital Grimsby, Grimsby); Ng HJ* (Dumfries and Galloway Royal Infirmary, Dumfries); Day A* (East Surrey Hospital, Redhill); Hunt A, Laskar N* (East Sussex Healthcare (Conquest Hospital and Eastbourne District General Hospital), Hastings); Gupta A*, Steinke J, Thrumurthy S (Epsom and St Helier University Hospitals NHS Trust, Epsom); Massie E, McGivern K, Rutherford D, Wilson M* (Forth Valley Royal Hospital, Larbert); Bacarese‐Hamilton T, Ip M*, James A, Salerno G*, Stockdale T (Frimley Health NHS FT—Wexham Park, Slough); Handa S, Kaushal M, Kler A, Patel P*, Redfern J, Tezas S (Furness General Hospital, Barrow In Furness); Aawsaj Y, Barry C, Blackwell L, Emerson H, Fisher A*, Katory M, Mustafa A (Gateshead Health NHS Foundation Trust, Gateshead); Kretzmer L*, Lalou L, Manku B, Parwaiz I, Stafford J (George Eliot Hospital, Nuneaton); Abdelkarim M, Asqalan A, Gala T, Ibrahim S, Maw A*, Mithany R, Morgan R*, Sundaram Venkatesan G (Glan Clwyd Hospital, Rhyl); Banfield D, Boal M*, Brown O, Dean H (Gloucestershire Royal Hospital, Gloucester); Boulton AJ (Good Hope Hospital, Sutton Coldfield); Hardie CM, McNaught C* (Harrogate District Hospital, Harrogate); Karandikar S*, Naumann D (Heartlands Hospital, Birmingham); Chen F, Cheung J* (Hinchingbrooke Hospital, Huntingdon); Ayorinde J, Chase T, Cuming T, Ghanbari A, Humphreys L, Tayeh S* (Homerton University Hospital, London); Aboelkassem Ibrahim A, Evans C, Ikram H, Loubani M*, Nazir S, Robinson A, Sehgal T, Wilkins A (Hull University Teaching Hospitals NHS Trust, Hull); Dixon J*, Jha M, Thulasiraman SV, Viswanath Y* (James Cook University Hospital, Middlesbrough); Curl‐Roper T, Delimpalta C, Liao CCL*, Velchuru V, Westwood E (James Paget University NHS Foundation Trust Hospital, Great Yarmouth); Bond‐Smith G*, Mastoridis S, Tebala GD, Verberne C (John Radcliffe Hospital, Oxford University Hospitals NHS Trust, Oxford); Anscomb N*, Baldwin‐Smith R, Davies M, Grainger C, Haji A, Haq A, Nunoo‐Mensah JW, Rizk M (King’s College Hospital, London); Bhatti MI, Boyd‐Carson H, Elsey E, Gemmill E, Herrod P*, MohammedJibreel M, Lenzi E, Saafan T, Sapre D, Sian T, Watson N (King’s Mill Hospital, Sutton‐In‐Ashfield); Athanasiou A*, Burke J, Costigan F, Elkadi H, Johnstone J, Nahm C (Leeds Teaching Hospitals Trust, Leeds); Annamalai S, Ashmore C, Kourdouli A (Leicester Royal Infirmary, Leicester); Chean CS, Dharamavaram S, Kulkarni N, Pereira I, Shanthakunalan K, Srikumar B (Lincoln County Hospital, Lincoln); Askari A, Cirocchi N, Kudchadkar S, Patel K, Sagar J*, Talwar R* (Luton and Dunstable University Hospital, Luton); Abdalla M, Ismail O, Newton K, Stylianides N* (Manchester Royal Infirmary, Manchester); Aderombi A, Bajomo O, Beatson K, Garrett WV*, Ng V (Medway Hospital, Gillingham); Al‐Habsi R, Divya GS, Dixon F, Keeler BD* (Milton Keynes University Hospital, Milton Keynes); Egan RJ*, Fabre I, Harries R*, Li Z, Parkins K, Spencer N, Thompson D (Morriston Hospital Swansea, Swansea); Gemmell C, Grieco C, Hunt L* (Musgrove Park Hospital, Taunton); Mahmoud Ali F (Newcastle Upon Tyne Hospitals NHS Foundation Trust, Newcastle Upon Tyne); Seebah K, Shaikh I*, Sreedharan L, Youssef M* (Norfolk and Norwich University Hospital, Norwich); Shah J* (North Manchester General Hospital, Manchester); Baguley M, Heer B, Rogers M, Woods R* (Northampton General Hospital, Northampton); Mills SJ* (Northumbria Healthcare NHS Foundation Trust, Northumberland and Tyne and Wear); Sahnan K (Northwick Park Hospital, Harrow); Ahmed ME, Bukhari SI, Illingworth B, Kanthasamy S, Knights E, Ong SL, Pujari R, Tan KHM, Vanker R* (Peterborough City Hospital, Peterborough); Michel M, Patil S, Ravindran S, Sarveswaran J*, Scott L (Pinderfields Hospital, Wakefield); Khan J* (Queen Alexandra Hospital, Portsmouth); Bhangu A*, Cato LD, Kamal M, Kulkarni R, Parente A, Saeed S, Vijayan D (Queen Elizabeth Hospital Birmingham, Birmingham); English C, Evans J*, Fell A*, Halkias C, Merh R, Nikolaou S (Queen Elizabeth The Queen Mother Hospital Margate, Margate); Kaul S, Khan AH, Khan F, Mukherjee S*, Patel M, Sarigul M, Singh S (Queen’s Hospital Romford, Romford); Adiamah A, Brewer H, Chowdhury A*, Evans J, Humes D*, Jackman J, Koh A, Lewis‐Lloyd C, Oyende O, Reilly J, Worku D (Queens Medical Centre, Nottingham); Bisset C, Moug S* (Royal Alexandra Hospital, Paisley); Chadha R* (Royal Berkshire Hospital, Reading); Math S, Sarantitis I, Timbrell S, Vitone L* (Royal Blackburn Hospital, Blackburn); Faulkner G* (Royal Bolton Hospital, Farnworth); Brixton G, Findlay L, Majkowska A, Manson J*, Potter R (Royal Bournemouth Hospital, Bournemouth); Bhalla A*, Chia Z, Daliya P, Grimley E, Malcolm FL, Theophilidou E (Royal Derby Hospital, Derby); Daniels IR, Fowler GE, Massey LH, McDermott FD*, Rajaretnam N (Royal Devon and Exeter Hospital, Exeter); Angamuthu N, Chowdhury S, Gilliland J, Hart C, Knowles J, Mirnezami R, Varcada M* (Royal Free Hospital, Hampstead); Beamish AJ, Magowan D, Nassa H, Price C, Smith L, Solari F, Tang AM, Williams G* (Royal Gwent Hospital, Newport); Davies E*, Hawkin P, Raymond T, Ryska O (Royal Lancaster Infirmary, Morecambe); Baron R*, Gahunia S, McNicol F*, Russ J, Szatmary P, Thomas A (Royal Liverpool University Hospital, Liverpool); Jayasinghe JD, Knowles C, Ledesma FS, Minicozzi A*, Navaratne L, Ramamoorthy R, Sohrabi C, Thaha MA*, Venn M (Royal London Hospital, London); Atherton R*, Brocklehurst M, McAleer J, Parkin E* (Royal Preston Hospital, Preston); Aladeojebi A, Ali M, Gaunt A* (Royal Stoke University Hospital, Stoke‐On‐Trent); Hammer C, Stebbing J (Royal Surrey County Hospital, Guildford); Coomber E, Williams O (Royal Sussex County Hospital, Brighton); Bunni J, Fairhurst K*, Mitchell S, Richards S (Royal United Hospital Bath, Bath); Hraishawi I, McIlmunn C, McIntosh S* (Royal Victoria Hospital, Belfast, Belfast); Bhasin S, Bodla AS, Burahee A, Crichton A, Fossett R, Yassin N* (Royal Wolverhampton NHS Trust, Wolverhampton); Barlow C, Ding D, Foster J, Longstaff L (Salisbury NHS Foundation Trust, Salisbury); Brown SR*, Lee M, Newman T, Steele C (Sheffield Teaching Hospital NHS Foundation Trust, Sheffield); Baker A, Konstantinou C, Ramcharan S*, Wilkin RJW (South Warwickshire NHS Foundation Trust, Warwick); Colvin HV, Shakoor Z (Southampton General Hospital, Southampton); Lawday S, Lyons A* (Southmead Hospital, Bristol); Newman S (Southport and Formby District General Hospital, Southport); Chung E, Hagger R, Hainsworth A, Karim A, Owen H, Ramwell A, Williams K* (St George’s Hospital, London); Hall J (Stepping Hill Hospital, Stockport); Harris G, Royle T*, Watson LJ (Sunderland Royal Hospital, Sunderland); Asaad P, Brown B, Duff S*, Khan A, Moura F, Wadham B (The University Hospital of South Manchester, Manchester); Mccluney S, Parmar C*, Shah S (The Whittington Hospital, London); Babar MS*, Goodrum S, Whitmore H (Torbay and South Devon NHS Trust, Torquay); Balasubramaniam D*, Jayasankar B*, Kapoor S, Ramachandran A (Tunbridge Wells Hospital, Maidstone); Beech N, Chand M*, Green L, Kiconco H, McEwen R (University College London Hospital, London); Pereca J* (University Hospital Ayr, Ayr); Arumugam S, Ibrahim B*, Khan K* (University Hospital North Durham, Durham); Gash K, Gourbault L, Maccabe TA, Newton C* (University Hospitals Bristol NHS Foundation Trust, Bristol); Baig M, Bates H, Dunne N, Khajuria A, Ng V, Sarma DR, Shortland T, Tewari N* (University Hospitals Coventry and Warwickshire NHS Trusts, Coventry); Akhtar MA*, Brunt A, McIntyre J, Milne K, Rashid MM, Sgrò A, Stewart KE, Turnbull A (Victoria Hospital Kirkcaldy, Kirkcaldy); Gossedge G*, O’Donnell S, Oldfield F (Warrington and Halton Teaching Hospitals NHS Trust, Warrington); Aguilar Gonzalez M*, Talukder S* (West Suffolk Hospital, Bury St Edmunds); Eskander P, Hanna M, Olivier J* (Weston General Hospital, Weston‐Super‐Mare); Basnyat P*, Davis H, Montauban P, Shrestha A (William Harvey Hospital, Ashford); Magee C*, Powell S* (Wirral University Teaching Hospital, Wirral); Flindall I, Hanson A, Mahendran V (Worcestershire Royal Hospital, Worcester); Green S, Lim M, MacDonald L, Miu V, Onos L, Sheridan K, Young R* (York Teaching Hospitals NHS Trust, York); Alam F, Griffiths O, Houlden C, Kolli VS, Lala AK, Seymour Z* (Ysbyty Gwynedd, Bangor, North Wales).

United States of America: Consorti E, Gonzalez R, Kwan‐Feinberg R*, Liu T (Alta Bates Summit Medical Center (Sutter Health), San Francisco); Cooper Z*, Hirji S, Mahvi D, Okafor B, Roxo V, Salim A (Brigham and Women’s Hospital, Boston); Loehrer A*, Telma K, Wilson M (Dartmouth‐Hitchcock Medical Center, Lebanon, NH); Bokenkamp M, Haynes AB, Hill C, Leede E, McElhinney K, Olson KA, Riley C (Dell Seton Medical Center at the University of Texas, Austin); Bigelow B, Etchill EW*, Gabre‐Kidan A*, Jenny HE, Kent A, Ladd MR, Long C, Malapati H, Margalit A, Rapaport S, Rose J, Stevens K, Tsai L, Vervoort D, Yesantharao P (Johns Hopkins Hospital, Baltimore, MD); Klaristenfeld D* (Kaiser Permanente San Diego Medical Center, San Diego); Huynh KT (Kaiser Permanente West Los Angeles, Los Angeles); Kaafarani H*, Naar L, Qadan M (Massachusetts General Hospital, Boston, MA); Cha DE, Gleeson E, Horn C, Sarpel U* (Mount Sinai, New York); Bhama A (Rush University Medical Centre, Chicago, IL); Colling K*, Najarian M (Saint Mary’s Medical Center‐Essentia Health, Duluth); Azam M, Choudhry A*, Marx W (Suny Upstate University Hospital, Syracuse); Chokshi R, Glass N*, Tsui G (The University Hospital, Newark, NJ); Abel MK, Boeck M, Chern H, Kornblith LZ*, Nunez‐Garcia B, Ozgediz D, Sarin A, Varma MG (University of California, San Francisco (UCSF), San Francisco); Abbott D, Acher A, Aiken T, Barrett J, Foley E, Schwartz PB, Zafar SN* (University of Wisconsin, Madison); Hawkins AT*, Maiga A (Vanderbilt University Medical Center, Nashville, TN); Ruzgar NM, Sion M, Ullrich S (Yale New Haven Hospital, New Haven, CT).

Yemen: Al‐Naggar H*, Al‐Shehari M*, Almassaudi A, Alsayadi M, Alsayadi R, Shream S (Al‐Thawra Modern General Hospital, Sana’a).
