# Supplementary material for: The impact of surgical delay on resectability of colorectal cancer: An international prospective cohort study
Source: Colorectal Dis. 2022 Apr 24;24(6):708–26. doi: 10.1111/codi.16117 (PMC9322431; doi:10.1111/codi.16117)
Supplement: Supplementary file 1 — Appendix S1 [file CODI-24-708-s001.docx]

#

# Appendix S1: Supplementary figures and tables

# Supplementary Figure 1: Neoadjuvant therapy regimen by cancer location, in all operated patients receiving neoadjuvant treatment.


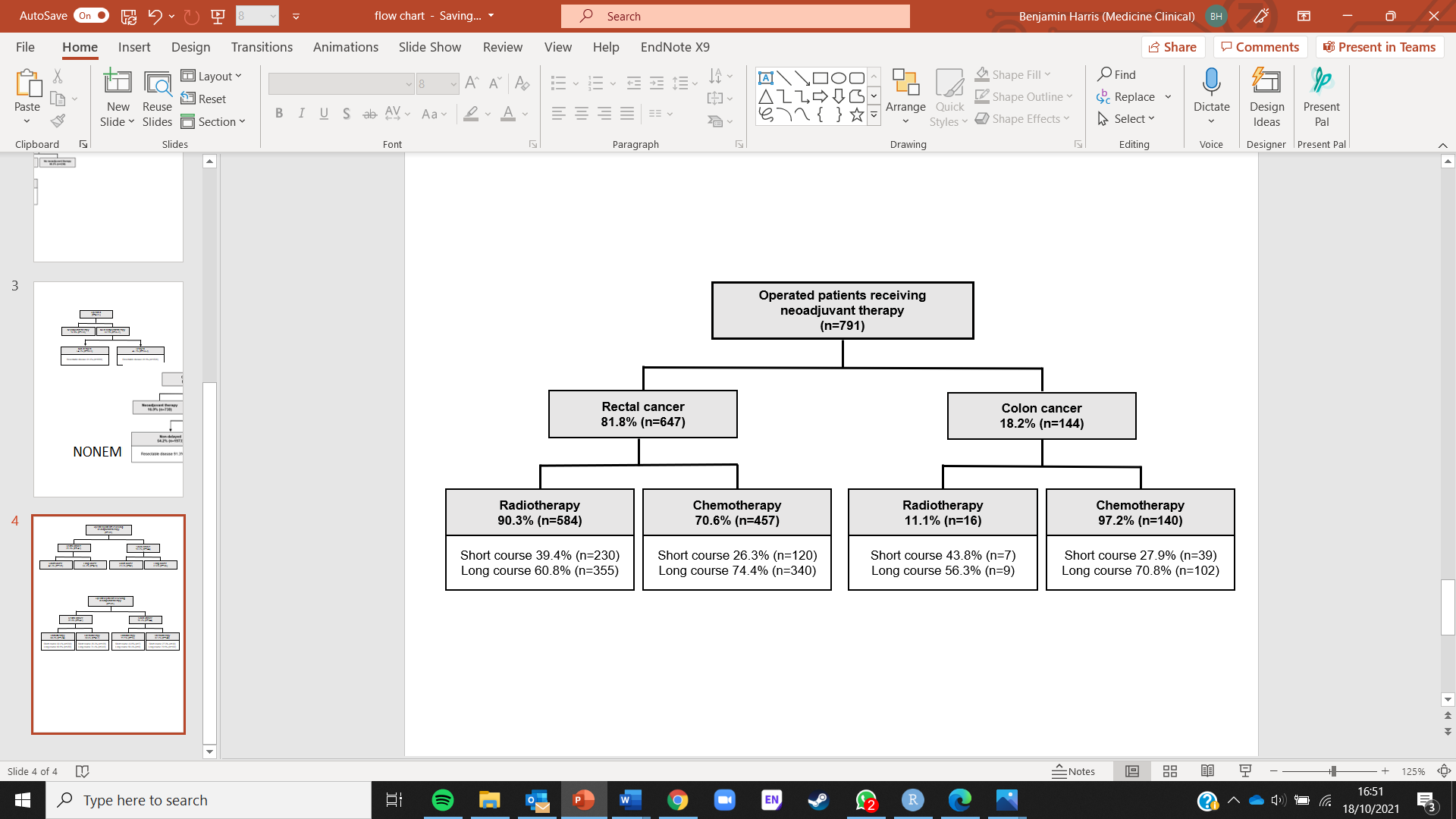


# Supplementary Figure 2: Multivariate logistic regression model exploring the association between delay to surgery and resectability for patients receiving elective surgery, adjusting for patient and disease factors.


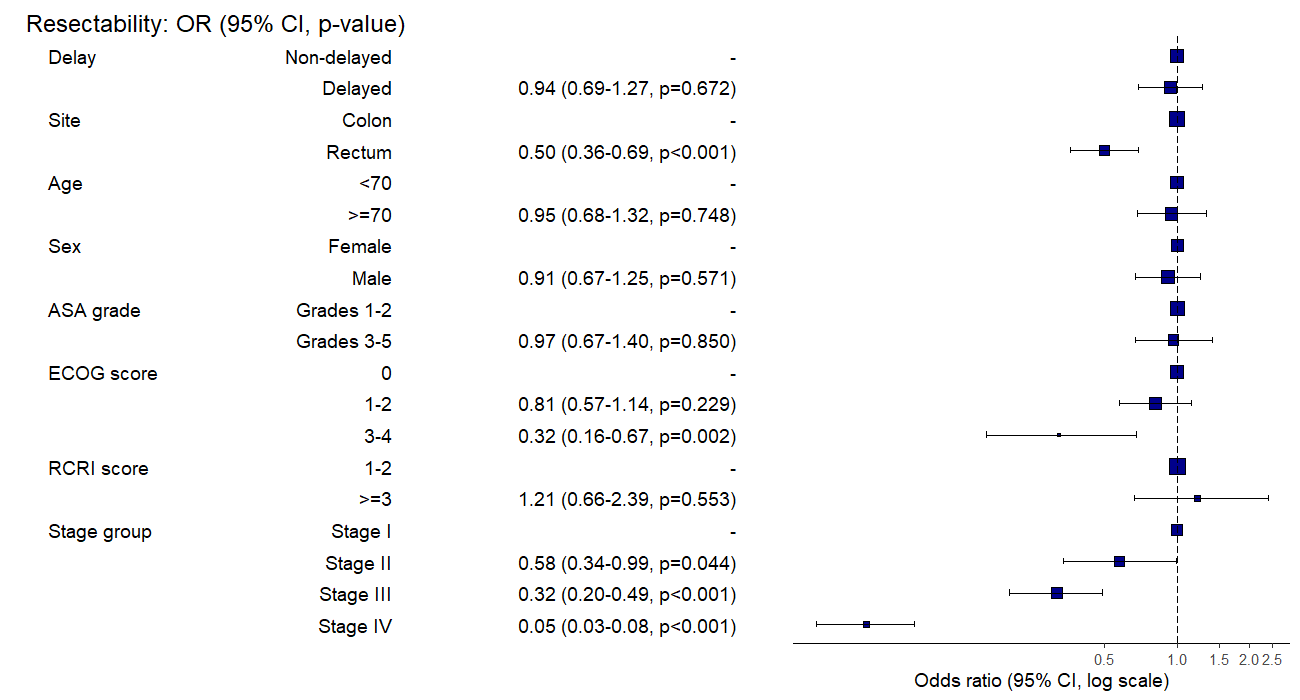


Number in dataframe = 3375, Number in model = 3375, Missing = 0, AIC = 1313.8, C-statistic = 0.768. Full model presented in Supplementary table 4. Delay was defined as a time from decision to treat to surgery of >4 weeks. Data reported as odds ratio (95% confidence interval, P value). OR>1 means higher odds of resectability for delayed patients, OR<1 means lower odds of resectability for delayed patients. ASA=American Society of Anaesthesiologists classification, ECOG=Eastern Cooperative Oncology Group, RCRI=Revised Cardiac Risk Index

# Supplementary Table 1: Demographic features of patients receiving and not receiving an operation.

|  | | **Operated**  **(n=5095)** | **Not Operated**  **(n=358)** | **P value** |
| --- | --- | --- | --- | --- |
| Site | Colon | 3446 (67.6) | 170 (47.5) | <0.001 |
|  | Rectum | 1649 (32.4) | 188 (52.5) |  |
| Age | <70 years | 2726 (53.5) | 209 (58.4) | 0.083 |
|  | ≥70 years | 2369 (46.5) | 149 (41.6) |  |
| Sex | Female | 2182 (42.8) | 145 (40.5) | 0.421 |
|  | Male | 2913 (57.2) | 213 (59.5) |  |
| ASA grade | 1-2 | 3413 (67.1) | 206 (60.1) | 0.009 |
|  | 3-5 | 1672 (32.9) | 137 (39.9) |  |
|  | Missing | 10 | 15 |  |
| ECOG Score | 0 | 2581 (51.3) | 135 (39.8) | <0.001 |
|  | 1-2 | 2298 (45.7) | 184 (54.3) |  |
|  | 3-4 | 148 (2.9) | 20 (5.9) |  |
|  | Missing | 68 | 19 |  |
| Revised Cardiac Risk Index | 1-2 | 4728 (92.8) | 327 (91.3) | 0.358 |
|  | ≥3 | 367 (7.2) | 31 (8.7) |  |
| BMI | Underweight | 174 (3.5) | 30 (9.3) | <0.001 |
|  | Normal | 2111 (42.3) | 120 (37.0) |  |
|  | Overweight | 1756 (35.1) | 111 (34.3) |  |
|  | Obese | 955 (19.1) | 63 (19.4) |  |
|  | Missing | 99 | 34 |  |
| Stage group | Stage I | 1572 (31.9) | 92 (26.4) | 0.004 |
|  | Stage II | 1044 (21.2) | 61 (17.5) |  |
|  | Stage III | 1808 (36.7) | 145 (41.5) |  |
|  | Stage IV | 507 (10.3) | 51 (14.6) |  |
|  | Missing | 164 | 9 |  |
| Country Income | High income | 4337 (85.1) | 265 (74.0) | <0.001 |
|  | Upper middle income | 472 (9.3) | 28 (7.8) |  |
|  | Low/lower-middle income | 286 (5.6) | 65 (18.2) |  |

Data reported as n (%). Percentages expressed of column total. P values calculated using Chi-square test. ASA=American Society of Anaesthesiologists classification, ECOG=Eastern Cooperative Oncology Group.

# Supplementary Table 2: Reasons why patients did not receive their operation.

|  | Number (%) (n=358) |
| --- | --- |
| Multidisciplinary team decision to cancel surgery due to patient risk | 260 (72.6) |
| Disease progression | 104 (29.1) |
| Patient unable to travel to hospital related to COVID-19 | 94 (26.3) |
| Patient choice to avoid surgery during COVID-19 pandemic | 82 (22.9) |
| Watch and wait surveillance only | 78 (21.8) |
| Performed local excision | 75 (20.9) |
| No bed/critical care bed/OR/theatre space available | 53 (14.8) |
| Change of recommendations in society guidelines related to COVID-19 | 38 (10.6) |
| Patient decision not related to COVID | 11 (3.1) |
| Delayed due to COVID-19 infection | 10 (2.8) |
| Collateral impact on services causing delay | 6 (1.7) |
| Other medical/surgical condition presented whilst waiting for surgery causing delay | 5 (1.4) |
| Patient unable to afford surgery | 3 (0.8) |
| Remission leading to decision not to operate | 2 (0.6) |
| Regression leading to change in plan and delay | 1 (0.3) |
| Awaiting restaging and/or surgical plan | 1 (0.3) |

Data reported as n (%). Patients could have had multiple of these reasons to not receive an operation.

# Supplementary Table 3: Reasons why patients received emergency surgery.

|  | Number (%) (n=663) |
| --- | --- |
| Gastrointestinal obstruction | 396 (59.7) |
| Tumour progression | 136 (20.5) |
| Bleeding | 100 (15.1) |
| Organ perforation | 64 (9.7) |
| Pain | 58 (8.7) |
| Sepsis | 28 (4.2) |
| Uncertain elective capacity | 2 (0.3) |

Data reported as n (%). Emergency surgery was any emergency surgery for colorectal cancer performed whilst awaiting the planned elective surgery, with or without resection.

# Supplementary Table 4: Full multivariate logistic regression model exploring the association between delay to surgery and resectability, adjusting for patient and disease factors.

|  | | **Non-resectable (n=297)** | **Resectable**  **(n=3669)** | **OR (univariable)** | **OR (multivariable)** |
| --- | --- | --- | --- | --- | --- |
| Delay | Non-delayed | 193 (8.2) | 2154 (91.8) | - | - |
|  | Delayed | 104 (6.4) | 1515 (93.6) | 1.31 (1.02-1.68, p=0.035) | 1.18 (0.90-1.55, p=0.224) |
| Site | Colon | 200 (6.6) | 2846 (93.4) | - | - |
|  | Rectum | 97 (10.5) | 823 (89.5) | 0.60 (0.46-0.77, p<0.001) | 0.51 (0.38-0.67, p<0.001) |
| Age | <70 years | 158 (7.9) | 1850 (92.1) | - | - |
|  | ≥70 years | 139 (7.1) | 1819 (92.9) | 1.12 (0.88-1.42, p=0.358) | 1.03 (0.78-1.36, p=0.833) |
| Sex | Female | 127 (7.3) | 1604 (92.7) | - | - |
|  | Male | 170 (7.6) | 2065 (92.4) | 0.96 (0.76-1.22, p=0.749) | 0.92 (0.71-1.19, p=0.510) |
| ASA grade | 1-2 | 187 (7.1) | 2437 (92.9) | - | - |
|  | 3-5 | 110 (8.2) | 1232 (91.8) | 0.86 (0.67-1.10, p=0.226) | 0.99 (0.73-1.36, p=0.972) |
| ECOG grade | 0 | 129 (6.4) | 1874 (93.6) | - | - |
|  | 1-2 | 144 (7.9) | 1690 (92.1) | 0.81 (0.63-1.03, p=0.090) | 0.74 (0.55-0.99, p=0.041) |
|  | 3-4 | 24 (18.6) | 105 (81.4) | 0.30 (0.19-0.50, p<0.001) | 0.29 (0.17-0.53, p<0.001) |
| RCRI grade | 1-2 | 278 (7.6) | 3387 (92.4) | - | - |
|  | ≥3 | 19 (6.3) | 282 (93.7) | 1.22 (0.77-2.03, p=0.421) | 1.17 (0.70-2.07, p=0.558) |
| Stage group | Stage I | 39 (2.7) | 1418 (97.3) | - | - |
|  | Stage II | 35 (4.0) | 838 (96.0) | 0.66 (0.41-1.05, p=0.078) | 0.65 (0.40-1.03, p=0.066) |
|  | Stage III | 102 (7.8) | 1205 (92.2) | 0.32 (0.22-0.47, p<0.001) | 0.33 (0.23-0.49, p<0.001) |
|  | Stage IV | 121 (36.8) | 208 (63.2) | 0.05 (0.03-0.07, p<0.001) | 0.05 (0.03-0.07, p<0.001) |

Number in dataframe = 3966, Number in model = 3966, Missing = 0, AIC = 1786.9, C-statistic = 0.776. Delay was defined as a time from decision to treat to surgery of >4 weeks. Data reported as odds ratio (95% confidence interval, P value). OR>1 means higher odds of resectability for delayed patients, OR<1 means lower odds of resectability for delayed patients. ASA=American Society of Anaesthesiologists classification, ECOG=Eastern Cooperative Oncology Group, RCRI=Revised Cardiac Risk Index

# Supplementary Table 5: Demographic features of patients having delayed and non-delayed, elective surgery only.

|  | | **Non-delayed**  **(n=1973)** | **Delayed**  **(n=1663)** | **P value** |
| --- | --- | --- | --- | --- |
| Site | Colon | 1511 (76.6) | 1213 (72.9) | 0.013 |
|  | Rectum | 462 (23.4) | 450 (27.1) |  |
| Age | <70 years | 1050 (53.2) | 778 (46.8) | <0.001 |
|  | ≥70 years | 923 (46.8) | 885 (53.2) |  |
| Sex | Female | 886 (44.9) | 672 (40.4) | 0.007 |
|  | Male | 1087 (55.1) | 991 (59.6) |  |
| ASA grade | 1-2 | 1390 (70.6) | 1031 (62.1) | <0.001 |
|  | 3-5 | 579 (29.4) | 629 (37.9) |  |
|  | Missing | 4 | 3 |  |
| ECOG Score | 0 | 1118 (57.3) | 763 (46.7) | <0.001 |
|  | 1-2 | 784 (40.2) | 825 (50.5) |  |
|  | 3-4 | 49 (2.5) | 47 (2.9) |  |
|  | Missing | 22 | 28 |  |
| Revised Cardiac Risk Index | 1-2 | 1845 (93.5) | 1520 (91.4) | 0.019 |
|  | ≥3 | 128 (6.5) | 143 (8.6) |  |
| Body Mass Index | Underweight | 61 (3.2) | 44 (2.7) | <0.001 |
|  | Normal | 860 (44.5) | 607 (37.2) |  |
|  | Overweight | 670 (34.7) | 610 (37.4) |  |
|  | Obese | 341 (17.7) | 372 (22.8) |  |
|  | Missing | 41 | 30 |  |
| Stage group | Stage I | 697 (36.8) | 683 (42.3) | 0.002 |
|  | Stage II | 407 (21.5) | 354 (21.9) |  |
|  | Stage III | 641 (33.8) | 472 (29.2) |  |
|  | Stage IV | 151 (8.0) | 105 (6.5) |  |
|  | Missing | 77 | 49 |  |
| Country Income | High income | 1721 (87.2) | 1512 (90.9) | 0.002 |
|  | Upper middle income | 168 (8.5) | 102 (6.1) |  |
|  | Low/lower-middle income | 84 (4.3) | 49 (2.9) |  |
| Approach | Open | 786 (39.9) | 743 (44.7) | 0.01 |
|  | Minimally invasive | 1080 (54.8) | 829 (49.9) |  |
|  | Converted to open | 104 (5.3) | 89 (5.4) |  |
|  | Missing | 3 | 2 |  |
| Anastomosis | Yes (with defunctioning stoma) | 260 (13.5) | 181 (11.1) | <0.001 |
|  | Yes (without defunctioning stoma) | 1413 (73.2) | 1152 (70.4) |  |
|  | No | 258 (13.4) | 304 (18.6) |  |
|  | Missing | 42 | 26 |  |
| Anastomotic method | Stapled | 1373 (82.2) | 1084 (81.4) | 0.613 |
|  | Handsewn | 298 (17.8) | 248 (18.6) |  |
|  | Missing | 302 | 331 |  |

Delay was defined as a time from decision to treat to surgery of >4 weeks. Data reported as n (%). Percentages expressed of column total. P values calculated using Chi-square test. ASA=American Society of Anaesthesiologists classification, ECOG=Eastern Cooperative Oncology Group.

# Supplementary Table 6: Multivariate logistic regression model exploring the association between delay to surgery and resectability in colon cancer patients, adjusting for patient and disease factors.

|  | | **Non-resectable (n=200)** | **Resectable**  **(n=2846)** | **OR (univariable)** | **OR (multivariable)** |
| --- | --- | --- | --- | --- | --- |
| Delay | Non-delayed | 142 (7.6) | 1720 (92.4) | - | - |
|  | Delayed | 58 (4.9) | 1126 (95.1) | 1.60 (1.18-2.21, p=0.003) | 1.33 (0.95-1.87, p=0.101) |
| Age | <70 years | 100 (6.8) | 1375 (93.2) | - | - |
|  | ≥70 years | 100 (6.4) | 1471 (93.6) | 1.07 (0.80-1.43, p=0.645) | 1.06 (0.75-1.48, p=0.753) |
| Sex | Female | 93 (6.8) | 1276 (93.2) | - | - |
|  | Male | 107 (6.4) | 1570 (93.6) | 1.07 (0.80-1.43, p=0.647) | 0.95 (0.70-1.30, p=0.767) |
| ASA grade | 1-2 | 121 (6.2) | 1842 (93.8) | - | - |
|  | 3-5 | 79 (7.3) | 1004 (92.7) | 0.83 (0.62-1.12, p=0.228) | 0.94 (0.65-1.37, p=0.740) |
| ECOG grade | 0 | 78 (5.3) | 1400 (94.7) | - | - |
|  | 1-2 | 104 (7.1) | 1354 (92.9) | 0.73 (0.53-0.98, p=0.038) | 0.69 (0.48-0.98, p=0.041) |
|  | 3-4 | 18 (16.4) | 92 (83.6) | 0.28 (0.17-0.51, p<0.001) | 0.33 (0.17-0.66, p=0.001) |
| RCRI grade | 1-2 | 184 (6.6) | 2618 (93.4) | - | - |
|  | ≥3 | 16 (6.6) | 228 (93.4) | 1.00 (0.61-1.76, p=0.995) | 1.00 (0.56-1.88, p=0.999) |
| Stage group | Stage I | 24 (2.2) | 1061 (97.8) | - | - |
|  | Stage II | 21 (3.0) | 682 (97.0) | 0.73 (0.41-1.34, p=0.308) | 0.77 (0.42-1.41, p=0.390) |
|  | Stage III | 63 (6.3) | 941 (93.7) | 0.34 (0.21-0.54, p<0.001) | 0.36 (0.22-0.58, p<0.001) |
|  | Stage IV | 92 (36.2) | 162 (63.8) | 0.04 (0.02-0.06, p<0.001) | 0.04 (0.03-0.07, p<0.001) |

Number in dataframe = 3046, Number in model = 3046, Missing = 0, AIC = 1227.6, C-statistic = 0.784. Delay was defined as a time from decision to treat to surgery of >4 weeks. Data reported as odds ratio (95% confidence interval, P value). OR>1 means higher odds of resectability for delayed patients, OR<1 means lower odds of resectability for delayed patients. ASA=American Society of Anaesthesiologists classification, ECOG=Eastern Cooperative Oncology Group, RCRI=Revised Cardiac Risk Index

# Supplementary Table 7: Multivariate logistic regression model exploring the association between delay to surgery and resectability in rectal cancer patients, adjusting for patient and disease factors.

|  | | **Non-resectable (n=97)** | **Resectable**  **(n=823)** | **OR (univariable)** | **OR (multivariable)** |
| --- | --- | --- | --- | --- | --- |
| Delay | Non-delayed | 51 (10.5) | 434 (89.5) | - | - |
|  | Delayed | 46 (10.6) | 389 (89.4) | 0.99 (0.65-1.52, p=0.977) | 0.91 (0.58-1.44, p=0.692) |
| Age | <70 years | 58 (10.9) | 475 (89.1) | - | - |
|  | ≥70 years | 39 (10.1) | 348 (89.9) | 1.09 (0.71-1.68, p=0.695) | 0.96 (0.59-1.56, p=0.857) |
| Sex | Female | 34 (9.4) | 328 (90.6) | - | - |
|  | Male | 63 (11.3) | 495 (88.7) | 0.81 (0.52-1.26, p=0.360) | 0.80 (0.50-1.27, p=0.356) |
| ASA grade | 1-2 | 66 (10.0) | 595 (90.0) | - | - |
|  | 3-5 | 31 (12.0) | 228 (88.0) | 0.82 (0.52-1.30, p=0.379) | 1.08 (0.62-1.94, p=0.783) |
| ECOG grade | 0 | 51 (9.7) | 474 (90.3) | - | - |
|  | 1-2 | 40 (10.6) | 336 (89.4) | 0.90 (0.58-1.41, p=0.650) | 0.88 (0.53-1.47, p=0.619) |
|  | 3-4 | 6 (31.6) | 13 (68.4) | 0.23 (0.09-0.69, p=0.005) | 0.18 (0.06-0.64, p=0.006) |
| RCRI grade | 1-2 | 94 (10.9) | 769 (89.1) | - | - |
|  | ≥3 | 3 (5.3) | 54 (94.7) | 2.20 (0.79-9.15, p=0.191) | 2.32 (0.74-10.32, p=0.197) |
| Stage group | Stage I | 15 (4.0) | 357 (96.0) | - | - |
|  | Stage II | 14 (8.2) | 156 (91.8) | 0.47 (0.22-1.00, p=0.048) | 0.47 (0.22-1.01, p=0.049) |
|  | Stage III | 39 (12.9) | 264 (87.1) | 0.28 (0.15-0.52, p<0.001) | 0.29 (0.15-0.53, p<0.001) |
|  | Stage IV | 29 (38.7) | 46 (61.3) | 0.07 (0.03-0.13, p<0.001) | 0.07 (0.03-0.13, p<0.001) |

Number in dataframe = 920, Number in model = 920, Missing = 0, AIC = 567.8, C-statistic = 0.745. Delay was defined as a time from decision to treat to surgery of >4 weeks. Data reported as odds ratio (95% confidence interval, P value). OR>1 means higher odds of resectability for delayed patients, OR<1 means lower odds of resectability for delayed patients. ASA=American Society of Anaesthesiologists classification, ECOG=Eastern Cooperative Oncology Group, RCRI=Revised Cardiac Risk Index

# Supplementary Table 8: Multivariate logistic regression model exploring the association between delay to surgery and resectability in patients with early disease, adjusting for patient and disease factors.

|  | | **Non-resectable (n=49)** | **Resectable**  **(n=2006)** | **OR (univariable)** | **OR (multivariable)** |
| --- | --- | --- | --- | --- | --- |
| Delay | Non-delayed | 28 (2.5) | 1090 (97.5) | - | - |
|  | Delayed | 21 (2.2) | 916 (97.8) | 1.12 (0.63-2.01, p=0.697) | 1.20 (0.67-2.18, p=0.537) |
| Site | Colon | 28 (1.8) | 1513 (98.2) | - | - |
|  | Rectum | 21 (4.1) | 493 (95.9) | 0.43 (0.25-0.78, p=0.004) | 0.41 (0.23-0.74, p=0.003) |
| Age | <70 years | 22 (2.2) | 975 (97.8) | - | - |
|  | ≥70 years | 27 (2.6) | 1031 (97.4) | 0.86 (0.48-1.52, p=0.608) | 0.93 (0.50-1.71, p=0.805) |
| Sex | Female | 21 (2.4) | 837 (97.6) | - | - |
|  | Male | 28 (2.3) | 1169 (97.7) | 1.05 (0.58-1.85, p=0.874) | 1.01 (0.56-1.80, p=0.970) |
| ASA grade | 1-2 | 29 (2.2) | 1280 (97.8) | - | - |
|  | 3-5 | 20 (2.7) | 726 (97.3) | 0.82 (0.46-1.48, p=0.507) | 0.86 (0.44-1.70, p=0.651) |
| ECOG grade | 0 | 21 (1.9) | 1058 (98.1) | - | - |
|  | 1-2 | 26 (2.8) | 894 (97.2) | 0.68 (0.38-1.22, p=0.198) | 0.66 (0.34-1.27, p=0.213) |
|  | 3-4 | 2 (3.6) | 54 (96.4) | 0.54 (0.15-3.40, p=0.407) | 0.48 (0.12-3.28, p=0.367) |
| RCRI grade | 1-2 | 46 (2.5) | 1817 (97.5) | - | - |
|  | ≥3 | 3 (1.6) | 189 (98.4) | 1.59 (0.58-6.61, p=0.437) | 1.93 (0.66-8.25, p=0.292) |
| Stage group | Stage I | 30 (2.3) | 1286 (97.7) | - | - |
|  | Stage II | 19 (2.6) | 720 (97.4) | 0.88 (0.50-1.61, p=0.678) | 0.86 (0.48-1.56, p=0.603) |

Number in dataframe = 2055, Number in model = 2055, Missing = 0, AIC = 470.7, C-statistic = 0.634. Delay was defined as a time from decision to treat to surgery of >4 weeks. Advanced disease was defined as T4, N1/2 or M1 disease; early disease was all other stages. Data reported as odds ratio (95% confidence interval, P value). OR>1 means higher odds of resectability for delayed patients, OR<1 means lower odds of resectability for delayed patients. ASA=American Society of Anaesthesiologists classification, ECOG=Eastern Cooperative Oncology Group, RCRI=Revised Cardiac Risk Index

# Supplementary Table 9: Multivariate logistic regression model exploring the association between delay to surgery and resectability in patients with advanced disease, adjusting for patient and disease factors.

|  | | **Non-resectable (n=239)** | **Resectable**  **(n=1531)** | **OR (univariable)** | **OR (multivariable)** |
| --- | --- | --- | --- | --- | --- |
| Delay | Non-delayed | 158 (13.9) | 979 (86.1) | - | - |
|  | Delayed | 81 (12.8) | 552 (87.2) | 1.10 (0.83-1.47, p=0.516) | 1.11 (0.81-1.52, p=0.517) |
| Site | Colon | 165 (12.0) | 1212 (88.0) | - | - |
|  | Rectum | 74 (18.8) | 319 (81.2) | 0.59 (0.44-0.80, p=0.001) | 0.51 (0.37-0.71, p<0.001) |
| Age | <70 years | 131 (13.8) | 821 (86.2) | - | - |
|  | ≥70 years | 108 (13.2) | 710 (86.8) | 1.05 (0.80-1.38, p=0.732) | 1.02 (0.74-1.40, p=0.915) |
| Sex | Female | 104 (12.9) | 704 (87.1) | - | - |
|  | Male | 135 (14.0) | 827 (86.0) | 0.90 (0.69-1.19, p=0.476) | 0.91 (0.67-1.22, p=0.530) |
| ASA grade | 1-2 | 154 (12.7) | 1056 (87.3) | - | - |
|  | 3-5 | 85 (15.2) | 475 (84.8) | 0.81 (0.61-1.09, p=0.161) | 1.10 (0.77-1.58, p=0.594) |
| ECOG grade | 0 | 103 (12.1) | 749 (87.9) | - | - |
|  | 1-2 | 114 (13.5) | 733 (86.5) | 0.88 (0.66-1.18, p=0.398) | 0.76 (0.55-1.06, p=0.103) |
|  | 3-4 | 22 (31.0) | 49 (69.0) | 0.31 (0.18-0.54, p<0.001) | 0.24 (0.12-0.46, p<0.001) |
| RCRI grade | 1-2 | 226 (13.5) | 1443 (86.5) | - | - |
|  | ≥3 | 13 (12.9) | 88 (87.1) | 1.06 (0.60-2.02, p=0.848) | 1.16 (0.61-2.34, p=0.669) |
| Stage group | Stage II | 16 (11.9) | 118 (88.1) | - | - |
|  | Stage III | 102 (7.8) | 1205 (92.2) | 1.60 (0.89-2.73, p=0.099) | 1.83 (1.01-3.16, p=0.037) |
|  | Stage IV | 121 (36.8) | 208 (63.2) | 0.23 (0.13-0.40, p<0.001) | 0.25 (0.14-0.44, p<0.001) |

Number in dataframe = 1770, Number in model = 1770, Missing = 0, AIC = 1236.1, C-statistic = 0.743. Delay was defined as a time from decision to treat to surgery of >4 weeks. Advanced disease was defined as T4, N1/2 or M1 disease. Data reported as odds ratio (95% confidence interval, P value). OR>1 means higher odds of resectability for delayed patients, OR<1 means lower odds of resectability for delayed patients. ASA=American Society of Anaesthesiologists classification, ECOG=Eastern Cooperative Oncology Group, RCRI=Revised Cardiac Risk Index

# Supplementary Table 10: Demographic features of patients stratified by length of delay.

|  | | **0-4 weeks (n=2559)** | **5-8 weeks (n=1089)** | **9-12 weeks (n=384)** | **>12 weeks**  **(n=271)** | **P value** |
| --- | --- | --- | --- | --- | --- | --- |
| Site | Colon | 2028 (79.2) | 798 (73.3) | 289 (75.3) | 187 (69.0) | <0.001 |
|  | Rectum | 531 (20.8) | 291 (26.7) | 95 (24.7) | 84 (31.0) |  |
| Age | <70 years | 1374 (53.7) | 532 (48.9) | 167 (43.5) | 120 (44.3) | <0.001 |
|  | ≥70 years | 1185 (46.3) | 557 (51.1) | 217 (56.5) | 151 (55.7) |  |
| Sex | Female | 1162 (45.4) | 468 (43.0) | 150 (39.1) | 102 (37.6) | 0.014 |
|  | Male | 1397 (54.6) | 621 (57.0) | 234 (60.9) | 169 (62.4) |  |
| ASA grade | 1-2 | 1764 (69.1) | 696 (64.0) | 222 (58.0) | 166 (61.5) | <0.001 |
|  | 3-5 | 789 (30.9) | 392 (36.0) | 161 (42.0) | 104 (38.5) |  |
|  | Missing | 6 | 1 | 1 | 1 |  |
| ECOG Score | 0 | 1343 (53.1) | 528 (49.3) | 150 (39.6) | 117 (44.7) | <0.001 |
|  | 1-2 | 1101 (43.5) | 511 (47.7) | 216 (57.0) | 140 (53.4) |  |
|  | 3-4 | 85 (3.4) | 32 (3.0) | 13 (3.4) | 5 (1.9) |  |
|  | Missing | 30 | 18 | 5 | 9 |  |
| RCRI | 1-2 | 2382 (93.1) | 1010 (92.7) | 351 (91.4) | 237 (87.5) | 0.008 |
|  | ≥3 | 177 (6.9) | 79 (7.3) | 33 (8.6) | 34 (12.5) |  |
| BMI | Underweight | 92 (3.7) | 28 (2.6) | 8 (2.1) | 9 (3.5) | <0.001 |
|  | Normal | 1121 (44.7) | 414 (38.5) | 134 (35.7) | 86 (33.1) |  |
|  | Overweight | 858 (34.2) | 390 (36.3) | 150 (40.0) | 106 (40.8) |  |
|  | Obese | 437 (17.4) | 243 (22.6) | 83 (22.1) | 59 (22.7) |  |
|  | Missing | 51 | 14 | 9 | 11 |  |
| Stage group | Stage I | 806 (32.8) | 431 (40.8) | 170 (45.5) | 108 (41.1) | <0.001 |
|  | Stage II | 560 (22.8) | 241 (22.8) | 78 (20.9) | 46 (17.5) |  |
|  | Stage III | 863 (35.1) | 312 (29.5) | 104 (27.8) | 87 (33.1) |  |
|  | Stage IV | 230 (9.4) | 72 (6.8) | 22 (5.9) | 22 (8.4) |  |
|  | Missing | 100 | 33 | 10 | 8 |  |
| Country Income | High income | 2143 (83.7) | 987 (90.6) | 350 (91.1) | 234 (86.3) | <0.001 |
|  | Upper middle income | 259 (10.1) | 67 (6.2) | 22 (5.7) | 27 (10.0) |  |
|  | Low/lower-middle income | 157 (6.1) | 35 (3.2) | 12 (3.1) | 10 (3.7) |  |
| Approach | Open | 1203 (47.1) | 501 (46.0) | 161 (42.0) | 138 (51.1) | 0.299 |
|  | Minimally invasive | 1216 (47.6) | 535 (49.1) | 196 (51.2) | 119 (44.1) |  |
|  | Converted to open | 137 (5.4) | 53 (4.9) | 26 (6.8) | 13 (4.8) |  |
|  | Missing | 3 | 0 | 1 | 1 |  |
| Anastomosis | Yes (with defunctioning stoma) | 330 (13.1) | 116 (10.8) | 43 (11.4) | 40 (14.9) | 0.1 |
|  | Yes (without defunctioning stoma) | 1716 (68.3) | 756 (70.5) | 265 (70.5) | 166 (61.7) |  |
|  | No | 467 (18.6) | 200 (18.7) | 68 (18.1) | 63 (23.4) |  |
|  | Missing | 46 | 17 | 8 | 2 |  |
| Anastomotic method | Stapled | 1646 (80.5) | 714 (82.0) | 244 (79.2) | 167 (81.1) | 0.711 |
|  | Handsewn | 398 (19.5) | 157 (18.0) | 64 (20.8) | 39 (18.9) |  |
|  | Missing | 515 | 218 | 76 | 65 |  |

Delay was measured from decision to treat to surgery. The total of patients reported in the table is 4303 (missing data for length of delay n=1). Data reported as n (%). Percentages expressed of column total. P values calculated using Chi-square test. ASA=American Society of Anaesthesiologists classification, ECOG=Eastern Cooperative Oncology Group, RCRI=Revised Cardiac Risk Index.

# Supplementary Table 11: Unadjusted outcomes compared by length of delay to surgery.

|  | | **0-4 weeks (n=2559)** | **5-8 weeks (n=1089)** | **9-12 weeks (n=384)** | **>12 weeks**  **(n=271)** | **P value** |
| --- | --- | --- | --- | --- | --- | --- |
| Resectability | Complete resection | 2261 (91.9) | 993 (93.7) | 352 (94.9) | 238 (92.2) | 0.094 |
|  | Incomplete resection | 199 (8.1) | 67 (6.3) | 19 (5.1) | 20 (7.8) |  |
|  | Missing | 99 | 29 | 13 | 13 |  |
| Resection margins | Positive | 107 (4.4) | 48 (4.6) | 12 (3.3) | 14 (5.5) | 0.58 |
|  | Negative | 2310 (95.6) | 1004 (95.4) | 356 (96.7) | 239 (94.5) |  |
|  | Missing | 142 | 37 | 16 | 18 |  |
| Progression to unresectable disease | Yes | 127 (5.0) | 24 (2.2) | 8 (2.1) | 8 (3.0) | <0.001 |
|  | No | 2432 (95.0) | 1064 (97.8) | 376 (97.9) | 263 (97.0) |  |
|  | Missing | 0 | 1 | 0 | 0 |  |
| Stage change  (from baseline to pathology) | Downstaged | 393 (18.1) | 200 (20.9) | 82 (23.9) | 53 (23.3) | 0.007 |
|  | No change | 1236 (56.9) | 501 (52.4) | 166 (48.4) | 108 (47.6) |  |
|  | Upstaged | 543 (25.0) | 255 (26.7) | 95 (27.7) | 66 (29.1) |  |
|  | Missing | 387 | 133 | 41 | 44 |  |
| New metastatic disease | Yes | 229 (10.1) | 63 (6.4) | 18 (5.1) | 17 (7.2) | <0.001 |
|  | No | 2036 (89.9) | 919 (93.6) | 334 (94.9) | 219 (92.8) |  |
|  | Missing | 294 | 107 | 32 | 35 |  |
| 30-day mortality | Died | 56 (2.2) | 17 (1.6) | 3 (0.8) | 6 (2.2) | 0.21 |
|  | Alive | 2502 (97.8) | 1072 (98.4) | 381 (99.2) | 265 (97.8) |  |
|  | Missing | 1 | 0 | 0 | 0 |  |
| 30-day major postoperative complications | Yes | 251 (9.8) | 100 (9.2) | 33 (8.6) | 30 (11.1) | 0.69 |
|  | No | 2307 (90.2) | 989 (90.8) | 351 (91.4) | 241 (88.9) |  |
|  | Missing | 1 | 0 | 0 | 0 |  |
| Urgency | Emergency | 585 (22.9) | 53 (4.9) | 14 (3.7) | 11 (4.1) | <0.001 |
|  | Elective | 1973 (77.1) | 1035 (95.1) | 368 (96.3) | 260 (95.9) |  |
|  | Missing | 1 | 1 | 2 | 0 |  |

Delay was measured from decision to treat to surgery. The total of patients reported in the table is 4303 (missing data for length of delay n=1). Data reported as n (%). Percentages expressed of column total. P values calculated using Chi-square test.
